# Determining the charge distribution and the direction of bond cleavage with femtosecond anisotropic x-ray liquidography

**DOI:** 10.1038/s41467-022-28168-0

**Published:** 2022-01-26

**Authors:** Jun Heo, Jong Goo Kim, Eun Hyuk Choi, Hosung Ki, Doo-Sik Ahn, Jungmin Kim, Seonggon Lee, Hyotcherl Ihee

**Affiliations:** 1 Department of Chemistry and KI for the BioCentury, Korea Advanced Institute of Science and Technology (KAIST), Daejeon, 34141 Republic of Korea; 2 Center for Advanced Reaction Dynamics, Institute for Basic Science (IBS), Daejeon, 34141 Republic of Korea

**Keywords:** Reaction kinetics and dynamics, Chemical physics

## Abstract

Energy, structure, and charge are fundamental quantities characterizing a molecule. Whereas the energy flow and structure change in chemical reactions are experimentally characterized, determining the atomic charges of a molecule in solution has been elusive, even for a triatomic molecule such as triiodide ion, I_3_^−^. Moreover, it remains to be answered how the charge distribution is coupled to the molecular geometry; which I-I bond, if two I-I bonds are unequal, dissociates depending on the electronic state. Here, femtosecond anisotropic x-ray solution scattering allows us to provide the following answers in addition to the overall rich structural dynamics. The analysis unravels that the negative charge of I_3_^−^ is highly localized on the terminal iodine atom forming the longer bond with the central iodine atom, and the shorter I-I bond dissociates in the excited state, whereas the longer one in the ground state. We anticipate that this work may open a new avenue for studying the atomic charge distribution of molecules in solution and taking advantage of orientational information in anisotropic scattering data for solution-phase structural dynamics.

## Abstract (web summary)

Tracking the flow of charge in reacting molecules may provide key insight into reaction mechanisms, but is particularly challenging in liquid solutions. Here the authors, by analyzing the isotropic and anisotropic scattering signal in femtosecond time resolved X-ray liquidography, determine the charge localization and structural changes during photodissociation of the triiodide anion I_3_^-^

## Introduction

**Fig. 1:**
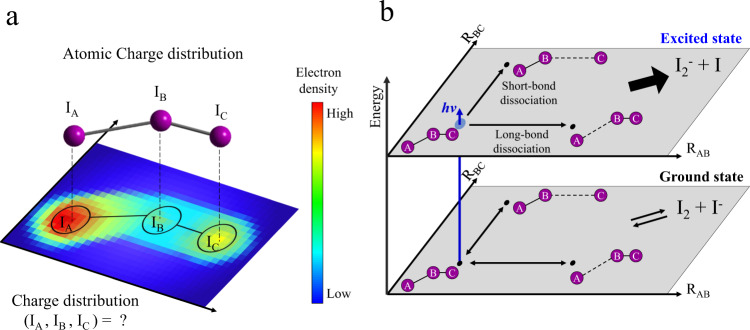
Fundamental questions on dynamics and structure of I_3_^−^. Simple yet fundamental questions on photodissociation dynamics of the triiodide ion in methanol such as **a** how the 1e^−^ charge of the ion is distributed among three iodine atoms, how the charge distribution is related with the geometry, and **b** which I–I bond, the shorter (R_BC_) or the longer one (R_AB_), is broken upon dissociation in the excited and ground states remain to be answered. In **a**, a case where the negative charge is localized on I_A_ is represented as an example. In **b**, two representative reaction pathways (short-bond dissociation and long-bond dissociation) of the two-body dissociation of I_3_^−^ are shown. See the main text for the details. In this work, time-resolved x-ray liquidography with the femtosecond time resolution was used to address these questions.

For understanding the reaction mechanism and reaction pathway of a chemical reaction, it is critical to track the change of the energy levels and three-dimensional structure in the reaction. Time-resolved spectroscopic techniques^1–9^ provide direct information on the energy flow based on the sensitivity to energy states, and time-resolved diffraction^10–25^ directly tracks the time-dependent structural change of a molecule based on the structural sensitivity. The charge distribution of a molecule or the flow of charge during a reaction plays a role as important as energy and structure, especially in reactions involving charge-transfer processes such as reactions in photoelectric and electrochemical devices^26,27^. Whereas the total charge or local charge distribution can be measured for molecules in solid or gas phases, no robust experimental method has yet been established to determine the atomic charges of individual atoms comprising a molecule in the liquid solution phase. For example, even for I_3_^−^, it has not yet been experimentally determined how the charge is distributed in each of the three I atoms in I_3_^−^ in the ground state^28–30^ (Fig. 1a). Moreover, although it is known that I_2_^−^ and I are formed by the excitation with 400 nm wavelength^31^, it is still elusive which specific bond dissociates among the two I–I bonds in I_3_^−^ (Fig. 1b). It also remains to be determined which bond cleavage is responsible for the equilibrium between I_3_^−^ and I^−^ + I_2_ in the ground state.

In a TRXL experiment, the x-ray pulse incident after the laser pulse at the time delay, Δ*t*, records the time evolution of the scattering patterns as well as the progress of the reaction and anisotropy. When a linearly polarized laser pulse interacts with an ensemble of molecules, the excitation probability of a molecule with a transition dipole, μ, is proportional to cos^2^$\phi$, where $\phi$ is the angle between the laser polarization (ε) and the transition dipole (μ). As a result, the orientational distribution of the excited molecules is transiently anisotropic at the moment of laser excitation^32^. Such photoselective alignment is apparent in the experimental difference scattering patterns at early time delays (Supplementary Fig. 1). The experimental scattering patterns were decomposed into isotropic and anisotropic components by considering the orientational distribution of photoselectively aligned molecules, and the resulting isotropic difference curves (ΔS_iso_) and anisotropic difference curves (ΔS_aniso_) were used for analysis to fully take advantage of the anisotropic information of the scattering patterns^33^. The ΔS_az_ (azimuthally averaged difference curves), ΔS_iso_, and ΔS_aniso_ are shown in Supplementary Fig. 2.

To address these questions, we used time-resolved x-ray liquidography (TRXL), also known as time-resolved x-ray solution scattering, and analyzed the anisotropic scattering signal contained in the TRXL data. Here, the challenge is to properly consider the anisotropy in the data analysis. Recently, a few studies focusing on ΔS_aniso_ beyond ΔS_iso_ have been reported^10,33^, but only the anisotropy for the solute term was analyzed, while the anisotropy of the solute-solvent (cage) and solvent terms was not incorporated in the analysis due to their relatively small contributions to the scattering patterns. In general cases, the anisotropies of all three terms (solute, cage, and solvent) need to be analyzed^34^, and accordingly, a proper method to extract the additional information from the anisotropy in the scattering patterns is required. In this work, we present an advanced data analysis considering the anisotropy of all the contributing terms of the TRXL signal.

## Results and discussion

### Ground state structure and charge distribution

The TRXL data measured at the XFEL has a significantly better signal-to-noise ratio (SNR) than the previously reported curve measured at the synchrotron^35^. The improved SNR turned out critical for further refining the molecular geometry of the ground-state I_3_^−^ and the atomic charge distribution, which could not be determined in the previous study. To determine the structure and charge distribution of I_3_^−^ in the ground state, a structural fitting analysis was performed using ΔS_az_ at 100 ps. Since I_2_^−^ and I are the only dominant species at 100 ps, the Debye scattering curves of I_3_^−^, I_2_^−^ and I were considered for calculating the solute term in the structural fitting analysis (see Methods section and Supplementary Fig. 5 for details). We also checked the sensitivity of the different scattering curve on the structure and charge distribution of I_3_^−^ by comparing cage terms calculated for several molecular geometries and charge distributions. As discussed in detail in Supplementary Information and Supplementary Fig. 4, the solute-solvent pair distribution function (PDF) is more sensitive to the atomic charge distribution than to the molecular geometry of I_3_^−^. The simulation results show that the charge distribution affects the solvent arrangement around the solute and accordingly alters the cage term in the TRXL signal, demonstrating the possibility of determining the charge distribution from the TRXL data based on the change of the cage term. In this regard, a recent TRXL study provided evidence that the difference in charge distribution affects the cage orientation, which was tracked by the change of the cage term, and revealed the direction of charge transfer in a FeRu complex with the aid of molecular dynamics (MD) simulations and time-dependent density functional theory calculations^36^. Here, we aimed to determine the quantitative atomic charges of individual atoms comprising a molecule purely based on the TRXL data.

**Fig. 2:**
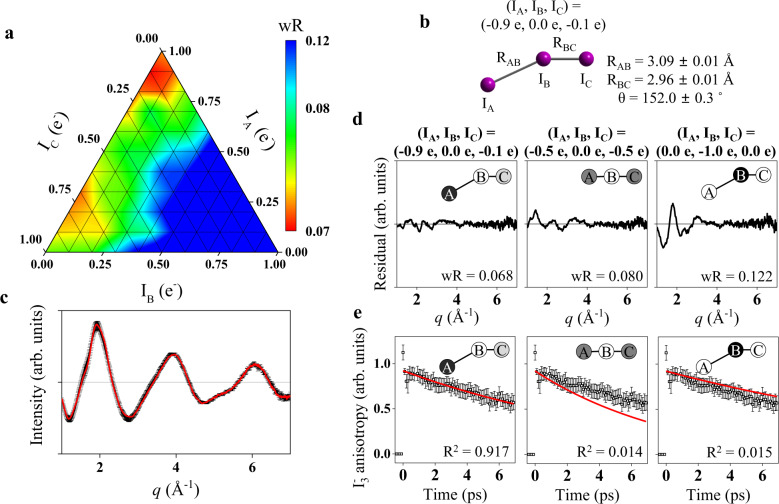
Determination of the charge distribution in I_3_^−^ in methanol. To determine the charge distribution, the data at 100 ps time delay was fitted using cage models calculated by MD simulations. The charge distribution of ground state I_3_^−^ was analyzed by screening all possible charge distributions. **a** The weighted R-factors (wRs) are presented as a function of the charges of three I atoms. The best fit, which gives the smallest wR, was obtained with (I_A_, I_B_, I_C_) = (−0.9 e, 0.0 e, −0.1 e). A color scale represents the wRs of each charge model. **b** The optimized structure of I_3_^−^ in methanol solvent. **c** The difference curve at 100 ps (black) with the corresponding standard error as error bars and the best fit curve (red) with (I_A_, I_B_, I_C_) = (−0.9 e, 0.0 e, −0.1 e). **d** Residual differences between the experimental curve and the fit curve were obtained for three representative cases of charge distributions. The atomic charges and the corresponding wRs are also shown for all three cases. (Left) The best fit case, which gives the smallest residual. (Middle) The case with the negative charge equally distributed on both I_A_ and I_C_ as determined from DFT calculation. (Right) The case with the negative charge is localized on I_B_. The magnitude of the atomic charge is schematically presented with white and black indicating zero and one full negative charge, respectively. The curves in **c** and **d** are on the same scale. **e** Comparison of the experimental anisotropy change (black dots with the corresponding standard deviations as error bars) and the calculated rotational correlation functions (RCFs) for the three representative cases of charge distributions shown in **d**. The coefficient of determination (R^2^) for each case is also shown. The best agreement, which gives the R^2^ value closest to 1, is found with the first case, providing additional support for the determined charge distribution. Error bars were obtained by independently fitting the individual experimental data. Source data for panel **c** are provided as a Source Data file.

The structural fitting analysis was performed by optimizing structural parameters of I_3_^−^ (bond lengths of I_A_−I_B_ (R_AB_) and I_B_−I_C_ (R_BC_), and I_A_−I_B_−I_C_ angle (θ)) and I_2_^−^ (the I–I bond length (R(I_2_^−^)), where the three iodine atoms of I_3_^−^ are labeled as I_A_, I_B,_ and I_C_, respectively. To consider the atomic charge distribution of I_3_^−^, new cage terms were generated by running MD simulations with I_3_^­−^ that have various charge distributions. Because MD simulations are slow processes, they could not be simply implemented into a maximum likelihood fitting procedure. To cope with this situation, we conducted grid search procedures by generating grid points of all possible atomic charge distributions. We considered negative atomic charges ranging from 0 to 1e^-^ and generated 66 grid points in total representing possible combinations of three atomic charges as shown in Fig. 2a and Supplementary Fig. 5. For each grid point, we performed the fitting iteratively until the solute structures to be optimized through the fitting process and those used to precalculate the cage term become self-consistent. The detailed procedure of the grid search is depicted in Supplementary Fig. 5. A plot of the weighted R-factors (wRs)^37,38^ for all grid points (that is, a grid map) shown in Fig. 2a visualizes that the charge distribution of (I_A_, I_B_, I_C_) = (−0.9 e, 0.0 e, −0.1 e) with an asymmetric bent structure (R_AB_ = 3.09 ± 0.01 Å, R_BC_ = 2.96 ± 0.01 Å and θ = 152 ± 0.4˚) yielded the smallest wR. More details of the resulting fitting parameters are summarized in Supplementary Table 1. The optimized atomic charge distribution indicates that the negative charge is (i) asymmetrically located. The majority of the negative charge is localized on the terminal I atom (I_A_) forming the longer I–I bond (I_A_−I_B_). We further inspected how the TRXL signal is altered depending on atomic charge distributions by comparing the fitting qualities at three representative grid points where the majority of the negative charge is (ii) equally distributed to I_A_ and I_C_, or (iii) localized on I_B_ (Fig. 2d). The comparison confirms that the various charge distributions are distinguishable from the TRXL data, and the different charge distributions predominantly affect the TRXL signal in a small-angle region (*q* < 3 Å^−1^).

As can be seen in Fig. 2a, a local minimum point arises at around (I_A_, I_B_, I_C_) = (−0.3 e, 0.0 e, −0.7 e), where the negative charge is localized on I_C_ forming the shorter I–I bond. Similar to the global minimum point, the local minimum point has an asymmetric charge distribution where the negative charge is localized on one of the terminal I atoms. The fact that both global and local minima are found at asymmetric charge distributions reflects that the TRXL signal, which is sensitive to the symmetry of charge distribution, supports an asymmetric charge distribution. The global minimum of (I_A_, I_B_, I_C_) = (−0.9 e, 0.0 e, −0.1 e) is more consistent with the consideration for the direction of bond cleavage, that is the shorter-bond dissociation vs the longer-bond dissociation, as well as the results of ab initio MD simulations and x-ray photoelectron spectroscopy, as discussed later. The possibility that the local minimum accounts for the actual minor charge distribution is less probable although it cannot be ruled out.

We also checked the possibility of overfitting or model bias by randomly choosing 10% of the data points, fitting only the remaining 90% of the data, and checking the weighted R-free factors (wR_free_). The wR_free_ is not significantly larger than the wR, indicating that the obtained fit result is sound. Moreover, another evidence supporting this finding is provided by considering the rotational dynamics, which turn out to depend on the charge distribution (Fig. 2d), as discussed later. Nevertheless, we cannot rule out the possibility that the exact global minimum may change if different MD simulations to obtain the cage term coupled with the charge distribution are used instead of the current classical MD simulations. The exploration in this regard is beyond the scope of the current study, but the approach outlined in this work should provide a road for such efforts.

The charge distribution obtained from the TRXL data can be compared with those obtained from previous theoretical studies. For example, the previous works using CASSCF calculation and the MD simulation suggested that the symmetry breaking of I_3_^−^ occurs along with the charge localization toward the terminal iodine under methanol solvent condition due to the strong solvent-solute interaction involving hydrogen bonds, although those studies did not provide information on whether the negative charge is localized at the terminal iodine forming the shorter bond or the longer bond^29,39^. A later report using ab initio simulation results finally showed that the I_3_^−^ in methanol has more negative charges on the terminal iodine forming the longer I–I bond^30^. Such charge localization toward the terminal iodine forming the longer bond was also reported for I_3_^−^ in an aqueous solution by an ab initio MD simulation^28^, suggesting that hydrogen bonds induce the symmetry breaking and charge localization on the relatively loosely-bounded terminal iodine forming the longer bond. As a result, the atomic charge distribution extracted from the TRXL data is in excellent agreement with the previous theoretical expectations, and the hydrogen bond between the hydroxyl group of methanol and I_3_^−^ can account for the symmetry breaking and charge localization observed in this work. The agreement between the results obtained from the theoretical calculations and our experiment demonstrates that TRXL bodes well for the experimental determination of the atomic charge distribution of molecules in solution. Furthermore, the symmetry breaking features of I_3_^−^ in terms of molecular geometry as well as the charge distribution obtained from TRXL are in line with those observed through Raman spectroscopy^40^ and x-ray photoelectron spectroscopy^28,30^. Specifically, the Raman signal corresponding to the antisymmetric stretching mode, which has to be symmetry forbidden in the linear geometry, was observed for I_3_^−^ in protic solvents such as water or ethanol while the signal is undetectable in acetonitrile, suggesting that I_3_^−^ is asymmetric in water or ethanol and symmetric in acetonitrile. Also, analysis of I(4d) core-level photoelectron spectra in conjunction with ab initio MD simulations support the symmetry breaking in I–I bond lengths with a more negative charge for the terminal iodine of the longer I–I bond while less negative charge for the terminal iodine of the shorter I–I bond, which is pronounced in water or methanol solvents^30^.

In addition to the charge distribution, the I_3_^−^ structures in methanol from this work can be compared with those from DFT calculations and previous studies. The calculated structure using a DFT method, which is presented in Supplementary Information, is symmetric linear I_3_^−^ with 2.95 Å bond length, which is in stark contrast with the optimized asymmetric structure from our work, 2.96 Å and 3.09 Å for two I–I bond lengths with an I–I–I angle of 152.0°. On the other hand, Raman spectroscopy^40^ and x-ray photoelectron spectroscopy^30^ provided evidence for asymmetric structure in protic solvents such as ethanol and water. Unlike the DFT structure, the structure using an ab initio MD simulation gives a good agreement with the TRXL structure^30^. Two I–I distances of the averaged structure of I_3_^−^ in methanol from an ab initio MD simulation (2.94 Å and 3.09 Å) agree well with those of the optimized structure (2.96 Å and 3.09 Å) although the I–I–I angle from the ab initio MD simulation (170.6°) is noticeably larger than that from the our TRXL data (152.0°).

### Determination of the direction of bond cleavage

**Fig. 3:**
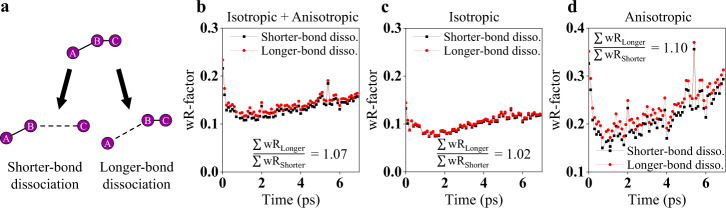
Determination of the direction of bond dissociation in I_3_^−^ in methanol. The fs-TRXL data were fitted with two different dissociation models. **a** Two models for bond dissociation, the shorter-bond dissociation and the longer-bond dissociation, are shown schematically. **b** The wRs obtained when the shorter-bond dissociation (black) or the longer-bond dissociation (red) model is used to fit the isotropic and anisotropic data, simultaneously. **c** The wRs for the isotropic data. **d** The wRs for the anisotropic data. The smaller wRs with the shorter-bond dissociation at all time delays in the anisotropic data support the shorter-bond dissociation over the longer-bond dissociation, whereas such a distinction is relatively less clear in the isotropic data. In **b**–**d**, the ratio of the sum of wRs for the longer-bond dissociation and that of the shorter-bond dissociation is also shown. The larger ratio indicates the better fit quality for the shorter-bond dissociation model relative to the longer-bond dissociation.

Regarding the photodissociation process of I_3_^−^, two possibilities were considered: whether the shorter I–I bond (I_B_−I_C_) or, the longer I–I bond (I_A_−I_B_) dissociates. To elucidate which model is more consistent with the experimental data, we performed structural analysis by fitting ΔS_iso_ and ΔS_aniso_ simultaneously with each of the two models. In the fitting, time-dependent fitting parameters include R(I_2_^−^), the distance between the I radical and the center of I_2_^−^ (r(I–I_2_^−^)), the root-mean-square displacement of the Debye-Waller factor (DWF) for r(I–I_2_^−^) (σ), and other parameters to account for temperature change, optical Kerr response of solvent, the population of I_2_^−^, and anisotropy (see “Structural analysis on ΔS_iso_ and ΔS_aniso_ (*q*, *t* > 300 fs)” and “Structural analysis on ΔS_iso_ and ΔS_aniso_ (*q*, *t* ≤ 300 fs)” in Supplementary Information for details). We introduced a DWF (exp(-σ^2^*q*^2^/2)) to r(I–I_2_^−^) to describe the relatively free movement of the escaping I radical, yielding a broad distribution of the interatomic distances (see Methods section for details). Figure 3b shows that at all time delays, the shorter-bond dissociation gives smaller wRs than the longer-bond dissociation, supporting that the shorter I–I bond of I_3_^−^ dissociates upon excitation. Then, we calculated the wRs for ΔS_iso_ and ΔS_aniso_, separately (Figs. 3c, d), to examine which one between ΔS_iso_ and ΔS_aniso_ is mainly responsible for the resolving power. Since ΔS_iso_ contains the information on interatomic distances only without any directional information, ΔS_iso_ alone is not expected to distinguish well between the two scenarios well. In contrast to ΔS_iso_, ΔS_aniso_ is more likely to provide a clue to the direction of bond cleavage since the two scenarios among the dissociation of the shorter or longer bond should yield different orientational distributions of the excited molecules, which eventually make distinct anisotropic scattering patterns. As can be seen in Fig. 3c, the wRs of ΔS_iso_ are not significantly different for both models whereas in ΔS_aniso_ the shorter-bond dissociation gives smaller wRs than the longer-bond dissociation at all time delays, confirming the prediction. The possibility of determining the direction of bond cleavage based on the anisotropic scattering signal was further confirmed by simulations of difference scattering curves arising from two dissociation models (see Supplementary Information for details).

The shorter-bond dissociation model has been suggested by several theoretical studies. For example, a study using an ab initio calculation showed that the dissociation of the shorter I–I bond is favored in the fragmentation of I_3_^−^ into I_2_^−^ and I^41^. An MD simulation study also showed that the shift of the peak position in the low *q* region of isotropic electron diffraction data is consistent with that of MD simulation with the assumption that the shorter-bond dissociation model^42^. We note that the direction of bond cleavage could be determined solely based on the TRXL signal without the aid of ab initio calculations or MD simulations. Meanwhile, the ground-state PES from the ab initio calculation shows that the formation of I_2_ and I^−^ is favored upon the dissociation of the longer I–I bond. The good agreement between TRXL data and the ab initio calculation for the excited state lends fidelity to the ab initio calculation for the ground state as well. If this is indeed the case, one can see that the bond dissociation and the charge distribution are coupled in such a way that the dissociation occurs in the direction where the redistribution of the atomic charge among atoms is minimized. In the ground state, where the formation of I_2_ and I^−^ is operational in the equilibrium, the longer, weaker I–I bond is likely to be broken unlike in the excited state. The different bond dissociations in the excited and ground states and the determined atomic charge distribution provide insight into how the atomic charge distribution is linked to which bond dissociates.

### Ultrafast structural dynamics of I_3_^−^

**Fig. 4:**
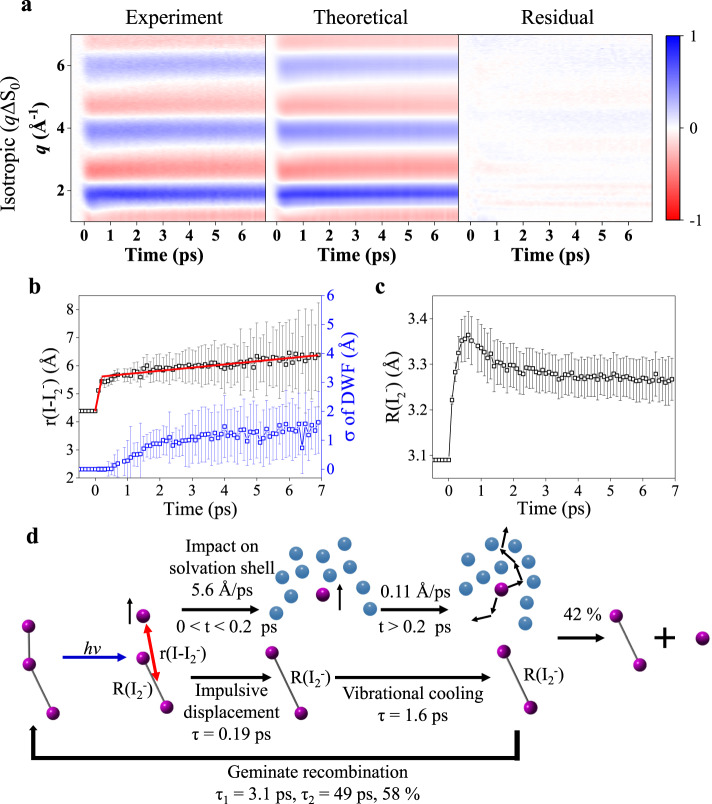
Structural dynamics from *q*ΔS_iso_ and the corresponding parameters and schematics on the dissociation dynamics of I_3_^−^. The experimental *q*ΔS_iso_ curves are fitted, and the corresponding structural parameters are presented. **a** Experimental *q*ΔS_iso_ (left), theoretical fits (middle), and the residual between the two (right) are presented (see Methods section for details). The individual curves and fits are also shown in Supplementary Fig. 14. All plots share a color scale representing the amplitude of the signal in absolute electronic units per solvent molecule. **b** r(I–I_2_^−^) and σ of the DWF with the corresponding standard deviations as error bars are presented. The r(I–I_2_^−^) and σ were used as fitting parameters. The r(I–I_2_^−^) shows two-phase velocity change induced by the intermolecular potential between I_2_^−^ and I radical and friction between the solvent molecule and I radical. Red lines represent linear fits of r(I–I_2_^−^) in the two phases. **c** The R(I_2_^−^) with the corresponding standard deviations as error bars are presented. **d** Schematics on the overall dynamics of I_3_^−^ photodissociation is shown. Upon 400 nm excitation, I_3_^−^ dissociates into I_2_^−^ and I radical. The dissociated I_2_^−^ undergoes impulsive displacement and vibrational cooling with time constants of 0.19 ps and 1.6 ps, respectively. The dissociated I radical moves away from I_2_^−^ with two phases in terms of the escape speed of I (5.6 and 0.11 Å/ps in the early and late time ranges, respectively). 58% of the dissociated fragments undergo geminate recombination to form the I_3_^−^ with two-time constants of 3.1 ps and 49 ps. Source data for panel **a** are provided as a Source Data file.

**Fig. 5:**
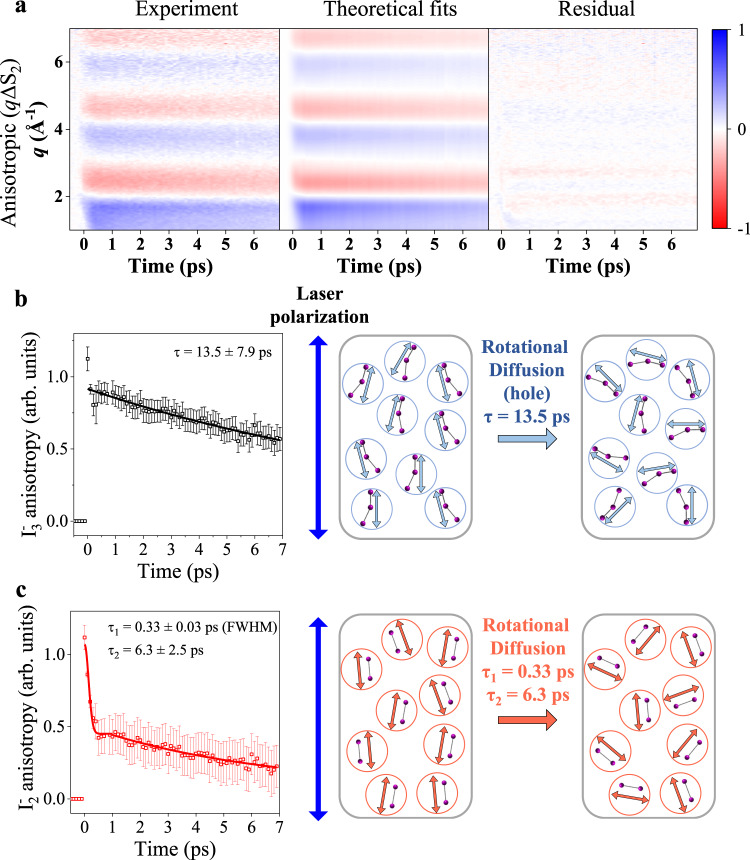
Structural dynamics from *q*ΔS_aniso_ and the corresponding parameters and schematics on the rotational dynamics of I_3_^−^ and I_2_^−^. **a** Experimentally acquired *q*ΔS_aniso_ (left), theoretical fits (middle), and the residual between the two (right) are presented (see Methods section for details). The individual curves and fits are also shown in Supplementary Fig. 14. All plots share a color scale representing the amplitude of the signal in absolute electronic units per solvent molecule. **b** The anisotropy coefficient of the I_3_^−^ with the standard deviations as error bars and corresponding rotational dynamics are presented. The anisotropy of I_3_^−^ originates from the holes of depleted I_3_^−^. This distribution is induced by a depleted portion of the I_3_^−^ which is selectively excited along the polarization axis of the pump laser. **c** The anisotropy coefficient of the I_2_^−^ with the standard deviations as error bars and corresponding rotational dynamics are presented. Because the dissociated I_2_^−^ is rotationally excited by the dissociation, I_2_^−^ shows the initial drop in the early time delay, which corresponds to the free rotor motion of the molecule. Source data for panel **a** are provided as a Source Data file.

Besides the bond cleavage direction, the structural analysis on both ΔS_iso_ and ΔS_aniso_, shown in Figs. 4a and 5a, respectively, unveils rich aspects of ultrafast structural dynamics such as the movement of the dissociating I and I_2_^−^, the escape dynamics of I, the vibrational relaxation, and rotational diffusion dynamics. In Fig. 4b, c, the time-dependent structural parameters, r(I–I_2_^−^), σ of the DWF for r(I–I_2_^−^), and R(I_2_^−^) are shown.

As shown in Fig. 4b, r(I–I_2_^−^) starts to increase right after the excitation and keeps increasing up to 7 ps, indicating that an I radical is formed by photoexcitation and the time-dependent r(I–I_2_^−^) represents the escape dynamics of I. Notably, the dynamics have two phases in terms of the escape speed of I, as shown in Fig. 4b. The I radical moves away with the higher speed of 5.6 ± 0.5 Å/ps in the early time range (0 fs < *t* < 200 fs), and the speed decreases by a factor of 52 down to 0.11 ± 0.01 Å/ps in the late time range (200 fs < *t* < 6.9 ps). This observation infers that the newly born I radical exhibits relatively free movement since the motion occurs within the solvent cage in the early time range, and then the movement of the radical becomes a diffusion-limited process through other solvent molecules after the escape of the original solvent cage^43^. This initial escaping speed of I is compatible with the result obtained from the time-resolved electron diffraction method aided by MD simulations, by which the dissociation speed of 5.8 Å/ps was determined^42^. In a recent study of the photodynamics of CH_2_I_2_ studied by TRXL^44^, similar to I_3_^−^, the rapid dissociation upon photoexcitation of I radicals was observed. The reported escape speed of the dissociated I radical is 2.1 Å/ps, which is slower than 5.6 Å/ps in I_3_^−^.

Similar to the time-dependent r(I–I_2_^−^), the time-dependent σ exhibit a two-phase rise in its temporal profile, as shown in Fig. 4b. In the early time range (0 fs < *t* < 200 fs), σ is almost zero, meaning that r(I–I_2_^−^) has a very narrow distribution in this time range. This observation can be attributed to the strong repulsive character of the excited-state PES of I_3_^−^ along the axis of the shorter I–I bond (R_BC_) as reported in a previous ab initio calculation^41^ performed on asymmetric I_3_^−^. Accordingly, the wavepacket moving in the dissociative pathway leading to the formation of an I radical is expected to be highly coherent, resulting in a small σ. In the late time range (200 fs < *t* < 6.9 ps), σ increases up to 1 Å. As mentioned in the discussion on r(I–I_2_^−^), the movement of the I radical in this late time range corresponds to the diffusion process after colliding with the solvent cage. The collision to the solvent cage can result in the loss of coherency of the moving wavepacket and thus induce the increase of σ, corresponding to the broader distribution of r(I–I_2_^−^).

The dynamics of I_2_^−^ generated by the escape of the I radical are reflected in R(I_2_^−^) (Fig. 4c). Similar to the time-dependent r(I–I_2_^−^), R(I_2_^−^) starts to increase right after the excitation. After 500 fs, R(I_2_^−^) decreases until reaching the equilibrium distance (3.25 Å). The initial elongation and subsequent contraction of R(I_2_^−^) occur with time constants of 0.19 ± 0.01 ps and 1.6 ± 0.2 ps, respectively. The initial increase of R(I_2_^−^) should arise from the initial motion of the vibrational wavepacket on the PES of I_2_^−^ starting from the Franck-Condon (FC) region. Since the shorter I–I bond (I_B_−I_C_) is dissociated upon excitation, the remaining I_A_−I_B_ converts into I_2_^−^ in the excited state. Considering that R_AB_ of I_3_^−^ in the ground state (3.09 Å) is shorter than the equilibrium R(I_2_^−^) (3.25 Å), the wavepacket generated in the FC region should initially move in the direction of increasing R(I_2_^−^). Consistent with this expectation, R(I_2_^−^) increases initially until 500 fs. The subsequent decrease of R(I_2_^−^) can be assigned to the vibrational relaxation to low-lying vibrational levels of the anharmonic PES of I_2_^−^, leading to the decrease of averaged I–I distance. The time constant of 1.6 ps assigned to the vibrational relaxation of I_2_^−^ is in good agreement with the previously reported values determined from spectroscopy experiments^43,45^. In Fig. 4d, the overall reaction dynamics of I_3_^−^ are summarized. In addition, the population dynamics of I_2_^−^ investigated in a wider time window from −2 ps to 100 ps indicate the existence of the geminate recombination of I_2_^−^ and I into I_3_^−^ with two-time constants of 3.1 ± 0.6 ps and 49 ± 22 ps and the fraction of the geminate recombination of 58 ± 11%, as shown in Supplementary Fig. 10.

### Rotational dephasing dynamics of I_3_^−^ and I_2_^−^

Time-dependent anisotropy of I_3_^−^ and I_2_^−^ obtained from the structural analysis are shown in Fig. 5b, c, respectively. In Fig. 5b, time-dependent anisotropy of the hole of depleted I_3_^−^ and the rotational diffusion dynamics corresponding to this anisotropy change are shown. The time-dependent anisotropy was fitted with a time constant of 13.5 ± 7.9 ps, corresponding to the rotational diffusion process in which the cos^2^$\phi$ orientational distribution of the ground-state hole relaxes to the isotropic distribution. In Fig. 5c, anisotropy of I_2_^−^ formed in the excited state and the corresponding rotational diffusion dynamics are shown. The change in the anisotropy of I_2_^−^, also arises from the relaxation of the cos^2^$\phi$ distribution to the isotropic orientational distribution of the excited-state molecules. Notably, unlike I_3_^−^, the temporal profile of anisotropy of I_2_^−^ exhibits two phases of the decay, including the rapid drop in the early time range (0 ps < *t* < 1 ps) and the subsequent slow decrease in the late time range (*t* > 1 ps)^45,46^, which can be attributed to the initial free rotor motion and subsequent rotational diffusion dynamics of I_2_^−^. The initial free rotor motion in the first phase (0 ps < *t* < 1 ps) can be fitted with a Gaussian function, yielding a similar full width at half maximum (FWHM) of 0.33 ± 0.03 ps to one in the previous study (Fig. 5c)^45^. The anisotropy of I_2_^−^ in the second phase (*t* > 1 ps) was fitted with an exponential function with a time constant of 6.3 ± 2.5 ps, which corresponds to the rotational diffusion process of I_2_^−^. The two time constants (13.5 ps and 6.3 ps) observed in the TRXL data are consistent with 15 ps and 5 ps assigned to the rotational diffusion time constants of I_3_^−^ and I_2_^−^, respectively, in a spectroscopic study^46^.

The rotational diffusion time constants of I_3_^−^ and I_2_^−^ extracted from the experimental data were also compared with those from rotational correlation functions (RCFs) calculated using the MD snapshots used to calculate the cage terms^47^. The optimized structure (R_AB_ = 3.09 Å, R_BC_ = 2.96 Å and θ = 152°) and charge distribution ((I_A_, I_B_, I_C_) = (−0.9 e, 0.0 e, −0.1 e)) of I_3_^−^ were used for this calculation. The resulting RCFs of I_3_^−^ and I_2_^−^ are shown in Supplementary Fig. 11. Both the RCFs of I_3_^−^ and I_2_^−^ were fitted with an exponential function, yielding time constants of 15.8 ± 0.2 ps and 3.8 ± 0.1 ps, respectively, showing good agreement with those extracted from the experimental data (13.5 ps for I_3_^−^ and 6.3 ps for I_2_^−^). It is noteworthy that RCFs change significantly depending on the charge distributions of I_3_^−^. For example, RCFs obtained for the cases where (ii) the charge is distributed equally at the two terminal I atoms ((I_A_, I_B_, I_C_) = (−0.5 e, 0.0 e, −0.5 e)) and (iii) the charge is localized at the central I atom ((I_A_, I_B_, I_C_) = (0.0 e, −1.0 e, 0.0 e)) decay with time constants of 8.9 ps and 22 ps, respectively (Supplementary Fig. 11c, d). Both cases where incorrect charge distributions were used for RCF calculations yielded the temporal profiles of the RCF_S_ different from the experimental observations (Fig. 2e). Therefore, the fact that the RCF of I_3_^−^ is consistent with the time-dependent anisotropy of I_3_^−^ only when the charge distribution of I_3_^−^ is (I_A_, I_B_, I_C_) = (−0.9 e, 0.0 e, −0.1 e) lends strong, additional support to the finding that this charge distribution determined from the structural analysis is reliable. These results demonstrate that the methodology using the cage term and proper treatment of the anisotropy in TRXL data analysis, presented in this work, offers experimental and analytics approach for determining the atomic charge distribution of a molecule in solution and the directionality of bond dissociation.

## Methods

### TRXL experiments and time-resolved difference curves

TRXL experiments were performed at the XSS beamline of PAL-XFEL (the Pohang Accelerator Laboratory x-ray free-electron laser), using the experimental protocol in previous publications. The details are presented in the Supplementary Information. Two-dimensional scattering images were averaged and decomposed to give isotropic and anisotropic one-dimensional scattering curves, S_iso_(*q*,*t*) and S_aniso_(*q*,*t*) respectively, as a function of momentum transfer, *q*, and time delay, *t*, between the laser and x-ray pulses^32^. Time-resolved difference scattering curves, ΔS_iso_(*q*,*t*) and ΔS_aniso_(*q*,*t*), were generated by subtracting the reference data measured at –20 ps from the data at other time delays. Extracted *q*ΔS_iso_(*q*,*t*) and *q*ΔS_aniso_(*q*,*t*) are shown in Supplementary Figs. 2 and 14.

### Determining the molecular structure and atomic charge distribution of the ground state

To determine the structure and charge distribution of I_3_^−^ in the ground state, a structural fitting analysis was performed using ΔS_az_ at 100 ps. We considered Debye scattering curves of I_3_^−^, I_2_^−^, and I radical for calculating the solute term in the structural fitting analysis (see Fig. 1b). The cage term was calculated with the aid of MD simulations, and the solvent term was experimentally acquired in a separate experiment with a heating dye in methanol solution (see Supplementary Information and Supplementary Fig. 5 for details). We note that only theoretical ΔS_iso_ were considered for the structural fitting analysis since the contribution of ΔS_aniso_ is negligible at 100 ps as the rotational dephasing processes are completed before 100 ps, as can be seen in Supplementary Fig. 2.

The structural fitting analysis was performed by optimizing structural parameters of I_3_^−^ (bond lengths of I_A_−I_B_ (R_AB_) and I_B_−I_C_ (R_BC_), and I_A_−I_B_−I_C_ angle (θ)) and I_2_^−^ (the I–I bond length (R(I_2_^−^)), where the three iodine atoms of I_3_^−^ are labeled as I_A_, I_B,_ and I_C_, respectively, as depicted in Fig. 1a. The cage terms were prepared before the structural fitting analysis for specific molecular geometries and charge distributions. Here, the basic strategy of the structural fitting analysis is to perform the analysis iteratively until the solute structures to be optimized through the fitting process, and the structures used to precalculate the cage term become self-consistent. Initially, a cage term of I_3_^−^ was calculated by an MD simulation performed against the symmetric linear structure (R_AB_ = R_BC_ = 2.95 Å, θ = 180˚) and symmetric charge distribution ((I_A_, I_B_, I_C_) = (−0.5 e, 0 e, −0.5 e)), which is the optimized values from a density functional theory (DFT) calculation on I_3_^−^ (see the section “Density functional theory calculation” in Methods section for the details on the DFT calculation). The cage term of I_2_^−^ for the analysis was initially determined using the structure calculated from a DFT calculation (R(I_2_^−^) = 3.23 Å). Throughout the entire analysis, we used an atomic charge of −0.5 e for two iodine atoms of I_2_^−^ since it is evident that the negative charge is equally distributed to the two iodine atoms. Then, the structural fitting analysis was performed using these cage terms by optimizing the structures of I_3_^−^ and I_2_^−^. The structure of I_3_^−^ resulting from the initial structural fitting has an asymmetric bent structure (R_AB_ = 3.14 ± 0.01 Å, R_BC_ = 2.92 ± 0.01 Å, θ = 151.2 ± 0.3˚). The structure of I_2_^−^ was determined to be R(I_2_^−^) = 3.28 ± 0.01 Å. These resulting structures of I_3_^−^ and I_2_^−^ are not self-consistent with the structure used for the calculation of the cage term. Moreover, the asymmetric bent structure of I_3_^−^ raises the possibility that the one quantum of the negative charge is asymmetrically distributed over three iodine atoms, unlike the symmetric linear structure, where the charge should be symmetrically distributed.

To consider the atomic charge distribution of I_3_^−^, new cage terms were generated by running MD simulations with the asymmetric bent I_3_^−^ that have various charge distributions. Because MD simulations are slow processes, they could not be simply implemented into a maximum likelihood fitting procedure. To cope with this situation, we conducted grid search procedures by generating grid points of all possible atomic charge distributions. We considered negative atomic charges ranging from 0 to 1e^−^, so that we generated 66 grid points in total with a grid point representing a possible combination of three atomic charges as shown in Fig. 2a and Supplementary Fig. 5. For each grid point, which is a given charge distribution, we ran an MD simulation to obtain the corresponding cage term while the asymmetric bent structure of I_3_^−^ determined from the initial structure fitting was used. Details on the MD simulations are described in the section “Molecular dynamics simulations” of Methods. Once all cage terms for all grid points were ready, we performed a fitting procedure for each grid point that refines the solute structures. Subsequently, we performed an additional optimization of the cage term and solute structures to attain self-consistency. Cage terms were calculated again using the refined structures of I_3_^−^ and I_2_^−^ determined in the previous fitting procedure for all the grid points. Finally, using the refined cage terms, the solute structures were further optimized via structural fittings for the grid points. The detailed procedure of the grid search is depicted in Supplementary Fig. 5.

### Structural analysis on ΔS_iso_ and ΔS_aniso_ (*q*, *t* > 300 fs)

In the structural analysis, the agreement between the theoretical and experimental difference scattering curves was quantified by the weighted R-factor (wR), which is given by the following equation.

1 $${{{{{\rm{wR}}}}}}(t)=\sqrt{\frac{\mathop{\sum}\limits_{i}\frac{{\left(\Delta {{{{{{\mathrm{S}}}}}}}_{{{{{{{\mathrm{theory}}}}}}},{{{{{{\mathrm{iso}}}}}}}}\left({q}_{i},t\right)-\Delta {{{{{{\rm{S}}}}}}}_{{{{{{\rm{iso}}}}}}}\left({q}_{i},t\right)\right)}^{2}}{{({\sigma }_{{{{{{\rm{iso}}}}}}}({q}_{i},t))}^{2}}+\mathop{\sum}\limits_{i}\frac{{\left(\Delta {{{\rm{S}}}_{{{{\mathrm{theory}}}},{{{\mathrm{aniso}}}}}}\left({q}_{i},t\right)-\Delta {{{{{{\rm{S}}}}}}}_{{{{{{\rm{aniso}}}}}}}\left({q}_{i},t\right)\right)}^{2}}{{({\sigma }_{{{{{{\rm{aniso}}}}}}}({q}_{i},t))}^{2}}}{\mathop{\sum}\limits_{i}\frac{{\left(\Delta {{{{{{\rm{S}}}}}}}_{{{{{{\rm{iso}}}}}}}\left({q}_{i},t\right)\right)}^{2}}{{({\sigma }_{{{{{{\rm{iso}}}}}}}({q}_{i},t))}^{2}}+\mathop{\sum}\limits_{i}\frac{{\left(\Delta {{{{{{\rm{S}}}}}}}_{{{{{{\rm{aniso}}}}}}}\left({q}_{i},t\right)\right)}^{2}}{{({\sigma }_{{{{{{\rm{aniso}}}}}}}({q}_{i},t))}^{2}}}}$$

Here, ΔS_iso_ and ΔS_aniso_ are the experimentally measured isotropic and anisotropic difference scattering signals, respectively, ΔS_theory,iso_ and ΔS_theory,aniso_ are the theoretical difference scattering curves calculated during the fitting processes, and *σ*_iso_ and *σ*_aniso_ are the standard errors of ΔS_iso_ and ΔS_aniso_, respectively. The minimization of the wR was performed to refine the molecular structure using the MINUIT package written at CERN and the error analysis was performed by MINOS, a built-in algorithm in the MINUIT software^48^. Structural and anisotropic parameters were optimized by minimizing the wR. The structure of ground state I_3_^−^ was fixed to that determined from the analysis of the 100-ps data. The fitting was conducted for each time delay by constructing the theoretical scattering curves calculated using the following equations.

2 $${\Delta {{{{{\mathrm{S}}}}}}}_{{{{{{{\mathrm{theory}}}}}}},{{{{{{\mathrm{iso}}}}}}}}\left(q,t\right)={{{\mathrm{B}}}_{0}}(t)* \left({{{{{{\mathrm{S}}}}}}}_{{{{{{\mathrm{e}}}}}},{{{{{{\mathrm{iso}}}}}}}}\left(q,t\right)-{{{{{{\mathrm{S}}}}}}}_{{{{{{\mathrm{g}}}}}},{{{{{{\mathrm{iso}}}}}}}}\left(q,t\right)\right)+{{{\mathrm{B}}}_{1}}(t)* {\left.\frac{\partial S\left(q\right)}{\partial T}\right|}_{\rho }$$

3 $${\Delta {{{{{\mathrm{S}}}}}}}_{{{{{{{\mathrm{theory}}}}}}},{{{{{{\mathrm{aniso}}}}}}}}\left(q,t\right)=	\, {{{\mathrm{B}}}_{0}}(t)* \left({{{\mathrm{A}}}_{{{\mathrm{e}}}}}(t)* {{{{{{\mathrm{S}}}}}}}_{{{{{{\mathrm{e}}}}}},{{{{{{\mathrm{aniso}}}}}}}}\left(q,t\right)-{{{\mathrm{A}}}_{{{\mathrm{g}}}}}(t)* {{{{{{\mathrm{S}}}}}}}_{{{{{{\mathrm{g}}}}}},{{\mathrm{aniso}}}}\left(q,t\right)\right)\\ 	+{{{\mathrm{B}}}_{2}}(t)* \Delta {{{{{{\mathrm{S}}}}}}}_{{{{{{{\mathrm{Kerr}}}}}}}}$$

In these equations, S_e,iso_ and S_g,iso_ are the theoretical isotropic scattering curves of the products (I_2_^−^ and I) and the reactant (I_3_^−^), respectively*.* S_e,aniso_ and S_g,aniso_ are the theoretical anisotropic scattering curves of I_2_^−^ and I_3_^−^. These scattering curves, S_e,iso_, S_g,iso_, S_e,aniso_, and S_g,aniso_ are composed of the solute term and the cage term. ${\left.\frac{\partial S\left(q\right)}{\partial T}\right|}_{\rho }$ is the contribution of the solvent heating, which is measured in separate experiments using a heating dye^19,49,50^. ΔS_Kerr_ is the anisotropic difference scattering signal arising from the Kerr effect of solvent molecules. ${\left.\frac{\partial S\left(q\right)}{\partial T}\right|}_{\rho }$ and ΔS_Kerr_, used for the structural analysis, are shown in Supplementary Fig. 13. ΔS_Kerr_ was extracted from the anisotropic solvent heating data, which was obtained from a separate TRXL experiment on a heating dye (see the “Structural analysis on ΔS_iso_ and ΔS_aniso_ (*q*, *t* > 300 fs)” section of Supplementary Information for the details). B_0_(*t*), B_1_(*t*) and B_2_(*t*) reflect the population dynamics of I_2_^−^, and the intensity of the solvent heating signal, and the intensity of the solvent Kerr signal, respectively. The fitted results are presented in Figs. 4 and 5. The detailed procedure and equations to calculate S_e,iso_ and S_g,iso_ in Eq. (2) and S_e,aniso_ and S_g,aniso_ in Eq. (3) are described in the section “Structural analysis on ΔS_iso_ and ΔS_aniso_ (*q*, *t* > 300 fs)” of Supplementary Information.

We constructed the theoretical isotropic and anisotropic scattering curves following Eqs. (2) and (3), and minimized the discrepancy between the theoretical and experimental data. To do so, we minimized the wR given by Eq. (1), by optimizing several structural parameters. In detail, the I–I bond length (R(I_2_^−^)), the distance between the I radical and the center of I_2_^−^ (r(I–I_2_^−^)), the root-mean-square displacement of the Debye-Waller factor (DWF) for r(I–I_2_^−^) (σ) were used as fitting parameters. Here, the DWF (exp(-σ^2^*q*^2^/2)) was introduced to describe the relatively free movement of the escaping I radical, yielding a broad distribution of the interatomic distance between the I radical and the center of I_2_^−^ (see the “Structural analysis on ΔS_iso_ and ΔS_aniso_ (*q*, *t* > 300 fs)” section of Supplementary Information). Here, we note that R(I_2_^−^), r(I–I_2_^−^), σ, A_e_(*t*), A_g_(*t*), B_0_(*t*), B_1_(*t*), and B_2_(*t*) were used as the time-dependent fitting parameters. Among these parameters, R(I_2_^−^), r(I–I_2_^−^) and B_0_(*t*) were used for both isotropic and anisotropic fitting processes, σ and B_1_(*t*) were used for the isotropic curve fitting, and the others, A_e_(*t*), A_g_(*t*), and B_2_(*t*) were used for the anisotropic data.

### Structural analysis on ΔS_iso_ and ΔS_aniso_ (*q*, *t* ≤ 300 fs)

To obtain the transient structures at time delays shorter than the temporal width of the experimental IRF (*t* ≤ 300 fs), we performed a structural analysis considering the convolution of the molecular response with the IRF. Following the previously reported procedures^11,23^, an approach to fit the experimental curves within the experimental IRF (*t* ≤ 300 fs) was slightly modified from that used for the time delays larger than the experimental IRF, described in the section “Structural analysis on ΔS_iso_ and ΔS_aniso_ (*q*, *t* > 300 fs)” of Methods. In detail, the time-dependent parameters for the structures of I_2_^−^ and the dissociating I radical, σ of the DWF, anisotropy coefficients (A_e_(*t*), and A_g_(*t*)) and the population of I_2_^−^ (B_0_(*t*)) were modeled by a quartic polynomial function as follows:

4 $${{{{{\rm{x}}}}}}\left({{{{{\rm{t}}}}}}\right)=\mathop{\sum }\limits_{k=0}^{4}{a}_{4-k}{t}^{4-k}$$

where a_4-*k*_ is the coefficient of the polynomial function. The isotropic and anisotropic scattering curves for the solute signal in S_inst,iso_(*q*, *t*) and S_inst,aniso_(*q*, *t*) were calculated based on *x*(*t*) from which the molecular structures were constructed and the DWF for r(I–I_2_^−^) was calculated. Subsequently, the instantaneous theoretical difference scattering curves, ΔS_inst_(*q*, *t*), were calculated using the following equation:

5 $$\Delta {{{{{{\rm{S}}}}}}}_{{{{{{{\mathrm{inst}}}}}}},{{{{{{\mathrm{iso}}}}}}}}\left(q,t\right)={{{\mathrm{B}}}}_{0}\left(t\right)* \big[{{{{{{\mathrm{S}}}}}}}_{{{{{{\mathrm{e}}}}}},{{{{{{\mathrm{iso}}}}}}}}\left(q,t\right)-{{{{{{\mathrm{S}}}}}}}_{{{{{{\mathrm{g}}}}}},{{{{{{\mathrm{iso}}}}}}}}(q)\big]+{{{\mathrm{B}}}}_{1}(t)* {\left.\frac{\partial S\left(q\right)}{\partial T}\right|}_{\rho }$$

6 $$\Delta {{{{{{\rm{S}}}}}}}_{{{{{{{\mathrm{inst}}}}}}},{{{{{{\mathrm{aniso}}}}}}}}\left(q,t\right)={{{\mathrm{B}}}}_{0}\left(t\right)* \left[{{{{{{\mathrm{S}}}}}}}_{{{{{{\mathrm{e}}}}}},{{{{{{\mathrm{aniso}}}}}}}}\left(q,t\right){{{\mathrm{A}}}}_{{{\mathrm{e}}}}\left(t\right)-{{{{{{\mathrm{S}}}}}}}_{{{{{{\mathrm{g}}}}}},{{{{{{\mathrm{aniso}}}}}}}}(q){{{\mathrm{A}}}}_{{{\mathrm{g}}}}\left(t\right)\right]+{{{\mathrm{B}}}}_{2}(t)* {\Delta {{{{{\mathrm{S}}}}}}}_{{{{{{{\mathrm{Kerr}}}}}}}}$$

where the terms in Eqs. (5) and (6) are the same as those in Eqs. (2) and (3). The fraction of the heating (B_1_(*t*)) and Kerr (B_2_(*t*)) signals was determined by using the SANOD during the fitting process minimizing the residual of the fit^51^. B_0_(*t*), A_e_(*t*), and A_g_(*t*) were also modeled by quadratic polynomial functions and the coefficients of the polynomial functions were used as fitting parameters. Then, ΔS_inst,iso_(*q*, *t*) and ΔS_inst,aniso_(*q*, *t*) were convoluted by the experimental IRF, IRF(*t*), by the following equations.

7 $$\Delta {{{{{{\rm{S}}}}}}}_{{{{{{{\mathrm{theory}}}}}}},{{{{{{\mathrm{iso}}}}}}}}\left(q,t\le 300\,{fs}\right)=\Delta {{{{{{\mathrm{S}}}}}}}_{{{{{{{\mathrm{inst}}}}}}},{{{{{{\mathrm{iso}}}}}}}}\left(q,t\right)\,\bigotimes \,{{{{{{\mathrm{IRF}}}}}}}(t)$$

8 $$\Delta {{{{{{\rm{S}}}}}}}_{{{{{{{\mathrm{theory}}}}}}},{{{{{{\mathrm{aniso}}}}}}}}\left(q,t\le 300\,{fs}\right)=\Delta {{{{{{\mathrm{S}}}}}}}_{{{{{{{\mathrm{inst}}}}}}},{{{{{{\mathrm{aniso}}}}}}}}\left(q,t\right)\,\bigotimes \,{{{{{{\mathrm{IRF}}}}}}}(t)$$

Under the constraints where the polynomial functions smoothly connect the structure at 0 fs, which is the structure of the I_2_^−^ at Frank-Condon region, and the structures at 300 fs determined following the procedures described in “Structural analysis on ΔS_iso_ and ΔS_aniso_ (*q*, *t* > 300 fs)” of Methods section, the coefficients of the polynomial functions for each parameter were optimized by minimizing the wR, which is given by the following equation:

9 $${{{{{\rm{wR}}}}}}=\sqrt{\frac{\mathop{\sum}\limits_{j={{{{{{\mathrm{time}}}}}}}\,{{{{{{\mathrm{delay}}}}}}}}\mathop{\sum}\limits_{i}\,\left[\frac{{\left(\Delta {{{\mathrm{S}}}}_{{{{{{{\mathrm{theory}}}}}}},{{{{{{\mathrm{iso}}}}}}}}\left({q}_{i},{t}_{j}\right)-\Delta {{{{{{\mathrm{S}}}}}}}_{{{{{{\mathrm{iso}}}}}}}\left({q}_{i},{t}_{j}\right)\right)}^{2}}{{\left({\sigma }_{{{{{{\rm{iso}}}}}}}\left({q}_{i},{t}_{j}\right)\right)}^{2}}\,+\,\frac{{\left(\Delta {{{\mathrm{S}}}}_{{{{{{{\mathrm{theory}}}}}}},{{{{{{\mathrm{aniso}}}}}}}}\left({q}_{i},{t}_{j}\right)-\Delta {{{{{{\mathrm{S}}}}}}}_{{{{{{\mathrm{aniso}}}}}}}\left({q}_{i},{t}_{j}\right)\right)}^{2}}{{\left({\sigma }_{{{{{{\rm{aniso}}}}}}}\left({q}_{i},{t}_{j}\right)\right)}^{2}}\right]}{\mathop{\sum}\limits_{j={{{{{{\mathrm{time}}}}}}}\,{{{{{{\mathrm{delay}}}}}}}}\mathop{\sum}\limits_{i}\,\left[\frac{{\left(\Delta {{{{{{\rm{S}}}}}}}_{{{{{{\rm{iso}}}}}}}\left({q}_{i},{t}_{j}\right)\right)}^{2}\,}{{\left({\sigma }_{{{{{{\rm{iso}}}}}}}\left({q}_{i},{t}_{j}\right)\right)}^{2}}+\,\frac{\,{\left(\Delta {{{{{{\rm{S}}}}}}}_{{{{{{\rm{aniso}}}}}}}\left({q}_{i},{t}_{j}\right)\right)}^{2}}{{\left({\sigma }_{{{{{{\rm{aniso}}}}}}}\left({q}_{i},{t}_{j}\right)\right)}^{2}}\right]}}$$

where *σ*_iso_ and *σ*_aniso_ is the standard deviation of the isotropic and anisotropic difference scattering intensity at each *q* and *t*. Finally, the resultant ΔS_theory,iso_(*q*, *t* ≤ 300 fs) and ΔS_theory,aniso_(*q*, *t* ≤ 300 fs) were concatenated with ΔS_theory,iso_(*q*, *t* > 300 fs) and ΔS_theory,aniso_(*q*, *t* > 300 fs), respectably, giving rise to ΔS_theory,iso_ and ΔS_theory,aniso_ shown in Figs. 4a and 5a, respectively. The optimized *x*(*t*) values were concatenated with the corresponding parameters used to calculate the solute signal of ΔS_theory,iso_(*q*, *t* > 300 fs) and ΔS_theory,aniso_(*q*, *t* > 300 fs) and are represented in Figs. 4b, c and 5b, c. See the section “Structural analysis on ΔS_iso_ and ΔS_aniso_ (*q*, *t* ≤ 300 fs)” of Supplementary Information for more details.

### Anisotropic cage term

The change of solute structure is accompanied by the change of cage structure around the solute molecule. We have developed a methodology to calculate the anisotropic cage term for any desired distribution of the solute molecules. First, we defined unit angular vectors ($\vec{{{{{{{\boldsymbol{v}}}}}}}_{{{{{{\boldsymbol{m}}}}}}}}$), which point evenly spaced spherical coordinates on the unit sphere using the spiral methods^52^, as shown in Supplementary Fig. 6a. A unit angular vector indicates the direction of the corresponding orientation-dependent PDF, ${g}_{{jk}}(r,\vec{{{{{{{\boldsymbol{v}}}}}}}_{{{{{{\boldsymbol{m}}}}}}}})$, to construct. To calculate ${g}_{{jk}}(r,\vec{{{{{{{\boldsymbol{v}}}}}}}_{{{{{{\boldsymbol{m}}}}}}}})$, we identified the desired atoms (for example, *k*) of solvent molecules within the polyhedral cone of the expanded sphere and calculated the PDF for the cross pair of an atom of a solute molecule (for example, *j*) and the selected solvent atoms within each polyhedral cone specified by $\vec{{{{{{{\boldsymbol{v}}}}}}}_{{{{{{\boldsymbol{m}}}}}}}}$. The cage signal corresponding to the orientation-dependent PDF is given by the following equation.

10 $${{{{{{\mathrm{S}}}}}}}_{{{{{{{\mathrm{cage}}}}}}}}\left(\vec{{{{{{\boldsymbol{q}}}}}}},\vec{{{{{{{\boldsymbol{v}}}}}}}_{{{{{{\boldsymbol{m}}}}}}}}\right)=2* \mathop{\sum}\limits_{j}\mathop{\sum}\limits_{k}{f}_{j}\left(\left|\vec{{{{{{\boldsymbol{q}}}}}}}\right|\right){f}_{k}\left(\left|\vec{{{{{{\boldsymbol{q}}}}}}}\right|\right){n}_{k}\int \frac{4\pi {r}^{2}}{N}({g}_{{jk}}(r,\vec{{{{{{{\boldsymbol{v}}}}}}}_{{{{{{\boldsymbol{m}}}}}}}})-1){\cos }(r\vec{{{{{{\boldsymbol{q}}}}}}\,}\cdot \vec{{{{{{{\boldsymbol{v}}}}}}}_{{{{{{\boldsymbol{m}}}}}}}}){dr}$$

Then, the cage signal for a single solute molecule embedded in solvent molecules is calculated by summing all ${{{{{{\mathrm{S}}}}}}}_{{{{{{{\mathrm{cage}}}}}}}}\left(\vec{{{{{{\boldsymbol{q}}}}}}},\vec{{{{{{{\boldsymbol{v}}}}}}}_{{{{{{\boldsymbol{m}}}}}}}}\right)$, as follows.

11 $${{{{{{\mathrm{S}}}}}}}_{{{{{{{\mathrm{cage}}}}}}}}\left(\vec{{{{{{\boldsymbol{q}}}}}}}\right) 	=\mathop{\sum }\limits_{m=1}^{N}{{{{{{\mathrm{S}}}}}}}_{{{{{{{\mathrm{cage}}}}}}}}(\vec{{{{{{\boldsymbol{q}}}}}}},\vec{{{{{{{\boldsymbol{v}}}}}}}_{{{{{{\boldsymbol{m}}}}}}}})\\ 	=2* \mathop{\sum }\limits_{m}^{N}\mathop{\sum}\limits_{j}\mathop{\sum}\limits_{k}{f}_{j}\left(\left|\vec{{{{{{\boldsymbol{q}}}}}}}\right|\right){f}_{k}\left(\left|\vec{{{{{{\boldsymbol{q}}}}}}}\right|\right){n}_{k}\int \frac{4\pi {r}^{2}}{N}({g}_{{jk}}(r,\vec{{{{{{{\boldsymbol{v}}}}}}}_{{{{{{\boldsymbol{m}}}}}}}})-1){\cos }(r\vec{{{{{{\boldsymbol{q}}}}}}\,}\cdot \vec{{{{{{{\boldsymbol{v}}}}}}}_{{{{{{\boldsymbol{m}}}}}}}}){dr}$$

Using Eq. (11), we calculated the scattering pattern from the solute-solvent pair for a solute molecule with any orientation. Generally, a single MD snapshot cannot give a satisfactory cage signal due to the insufficient sampling of the positions and orientations of solvent molecules. To compensate for this issue, we calculated the averaged orientation-dependent PDFs extracted from the 200,000 MD snapshots as follows:

12 $${g}_{{jk}}^{{ave}}\left(r,\vec{{{{{{{\boldsymbol{v}}}}}}}_{{{{{{\boldsymbol{m}}}}}}}}\right)=\mathop{\sum }\limits_{i=1}^{200,000}{g}_{{jk}}^{(i)}\left(r,\vec{{{{{{{\boldsymbol{v}}}}}}}_{{{{{{\boldsymbol{m}}}}}}}}\right)$$

where ${g}_{{jk}}^{(i)}\left(r,\vec{{{{{{{\boldsymbol{v}}}}}}}_{{{{{{\boldsymbol{m}}}}}}}}\right)$ is the orientation-dependent PDF calculated from the *i*-th MD snapshot and ${g}_{{jk}}^{{ave}}\left(r,\vec{{{{{{{\boldsymbol{v}}}}}}}_{{{{{{\boldsymbol{m}}}}}}}}\right)$ is the averaged orientation-dependent PDFs over 200,000 MD snapshots (see Supplementary Fig. 7c). Using the averaged orientation-dependent PDFs (${g}_{{jk}}^{{ave}}\left(r,\vec{{{{{{{\boldsymbol{v}}}}}}}_{{{{{{\boldsymbol{m}}}}}}}}\right)$), the cage scattering pattern arising from the anisotropic orientation of the solute molecules was calculated as follows.

13 $${{{{{{\mathrm{S}}}}}}}_{{{{{{{\mathrm{cage}}}}}}}}{\prime} \left(\vec{{{{{{\boldsymbol{q}}}}}}}\right)=\mathop{\sum }\limits_{m}^{N}\mathop{\sum}\limits_{j}\mathop{\sum}\limits_{k}{f}_{j}\left(\left|\vec{{{{{{\boldsymbol{q}}}}}}}\right|\right){f}_{k}\left(\left|\vec{{{{{{\boldsymbol{q}}}}}}}\right|\right){n}_{k}\int \frac{4\pi {r}^{2}}{N}({g}_{{jk}}^{{ave}}(r,\vec{{{{{{{\boldsymbol{v}}}}}}}_{{{{{{\boldsymbol{m}}}}}}}})-1){\cos }(r\vec{{{{{{\boldsymbol{q}}}}}}\,}\cdot \vec{{{{{{{\boldsymbol{v}}}}}}}_{{{{{{\boldsymbol{m}}}}}}}}){dr}$$

So far, we explained how the cage scattering term for a single solute molecule with a certain fixed orientation embedded in a box of solvent molecules. The next step is to consider the effect of the anisotropic distribution induced by the photoselective alignment. The anisotropic distribution of excited molecules induced by a linearly polarized laser pulse is given by P($\phi$) ∝ cos^2^$\phi$, where $\phi$ stands for the angle between the laser polarization and the transition dipole moment^32^. To consider the effect of molecular orientations, we rotated each MD snapshot to retrieve the molecular orientations induced by the photoexcitation where the rotated solute molecules have the cos^2^ distribution, as shown in Supplementary Fig. 7b. For each of the rotated MD snapshots, the corresponding orientation-dependent PDFs were calculated following the procedures described above. Then, the orientation-dependent PDFs calculated from the rotated MD snapshots were averaged for each unit angular vector. Using the averaged orientation-dependent PDFs (${g}_{{jk}}^{{ave}}\left(r,\vec{{{{{{{\boldsymbol{v}}}}}}}_{{{{{{\boldsymbol{m}}}}}}}}\right)$), the cage scattering pattern arising from the anisotropic orientation of the solute molecules was calculated with Eq. (13). Finally, isotropic and anisotropic cage terms were extracted from ${{\mathrm{S}}}_{{{\mathrm{cage}}}}{\prime} \left(\vec{{{{{{\boldsymbol{q}}}}}}}\right)$ following the previously reported procedures^33^ as shown in Supplementary Fig. 8. More details are described in the section “Anisotropic cage term” of Supplementary Information.

### Density functional theory calculation

Geometry optimization was performed using density functional theory (DFT) for I_2_^−^ and I_3_^−^ to obtain the molecular structure for initial cage calculations. We used the recently-developed ωB97XD functional^53^ as a DFT exchange-correlation functional. To treat the scalar relativistic effect of iodine, we used aug-cc-pVDZ-PP small-core relativistic effective core potential (RECP)^54^. For other atoms (C, O, and H), 6- 31++G(d) basis sets were used. We also used the integral-equation-formalism polarizable continuum model (IEFPCM) method^55^ to describe the solvent effect implicitly. We used the natural population analysis (NPA) for characterizing atomic charge. All DFT calculations were performed using the Gaussian16 program.

### Molecular dynamics simulations

Molecular dynamics (MD) simulations were performed using the MOLDY program to obtain the cage terms for all the chemical species involved in the reaction^56^. One rigid solute molecule was embedded in a virtual cubic box of ~32.5 Å size containing 512 rigid methanol solvent molecules. The internal structure of each molecule was fixed, and the intermolecular interactions were governed by Coulomb forces and Lennard-Jones potentials. For describing the intermolecular interactions, we used a six-site all-atom (AA) model of methanol, OPLS-AA force field^57,58^. Structures of solute molecules (I_3_^−^, I_2_^−^, and I•) were optimized using the DFT method, as written in the section “Density functional theory calculation” of Methods. All simulations were performed at an ambient temperature of 300 K with a solvent density of 0.792 g/cm^3^. The system was equilibrated over 20 ps via coupling to a Nose-Hoover thermostat^59^. The simulations were performed in the NVT ensemble with a time step of 0.1 fs, and the trajectories were followed up to 1 ns.

### Rotational dynamics simulation

The analysis of the experimental data reveals that the value of the rotational diffusion time constants of I_3_^−^ and I_2_^−^ are 13.5 ps and 6.3 ps, respectively, as shown in Fig. 5. To support the experimental results, we conducted simulations to verify the rotational diffusion time constants of the intermediates^47,60^. The anisotropy at time *t*, *r*(*t*), measures the correlation function of the second Legendre polynomial of the scalar product polarization direction and the orientation of molecules^46^ (transition dipole moments) at time delay, *t*.

14 $${{{{r}}}}\left(t\right)=A \, < \, {P}_{2}\left[{\mu }_{{pump}}\,\left(0\right)\,{\mu }_{{ori}}\,\left(t\right)\right] > =A \, < \, {P}_{2}\,\left[{\cos }\theta \right] \, > $$

Here, $\theta$ is the angle between the pump polarization and the orientation of the molecule and A is the anisotropy of the molecule. Based on the equation above, we calculate the correlation function of I_3_^−^ and the I_2_^−^ by using the MD simulation assuming the orientation of the molecule is aligned to the polarization axis of the pump pulse. Details of the MD simulation is described in the section “Molecular dynamics simulations”, noting that the parameters of the MD simulation used for the cage calculation were used identically. From the simulation, snapshots were extracted every 10 fs, and the unit vector of transition dipole moment (same with the unit vector of dissociating bond for I_3_^−^) was calculated. Because the rotational diffusion dynamics of the molecule should occur regardless of the initial condition, we calculated the correlation function of the molecules for every single step of the simulation and averaged the simulated data. Calculated correlation functions according to Eq. (14) are presented in Supplementary Fig. 11. The rotational diffusion time constant of I_3_^−^ and I_2_^−^ are calculated to be 15.8 ps and 3.8 ps, respectively. The rotational diffusion time constants obtained from the simulation are consistent with those obtained from the analysis of the experimental data shown in Fig. 5 and those reported in previous studies^45,46^.

## Supplementary information

Supplementary information

## Data Availability

The time-resolved isotropic and anisotropic difference scattering data analyzed in this study corresponding to Figs. 2c, 4a and 5a are provided as the Source Data file. All relevant data that support the findings of this study are available from the corresponding author upon request. Source data are provided with this paper.

## Source data

Source Data

## Code availability

The codes used for the analysis here are available from the corresponding author on request.
