# Supplementary material for: Determining the charge distribution and the direction of bond cleavage with femtosecond anisotropic x-ray liquidography
Source: Nat Commun. 2022 Jan 26;13:522. doi: 10.1038/s41467-022-28168-0 (PMC8792042; doi:10.1038/s41467-022-28168-0)
Supplement: Supplementary file 1 — Supplementary information [file 41467_2022_28168_MOESM1_ESM.pdf]

Supplementary Information

Determining the charge distribution and the direction of bond cleavage with femtosecond anisotropic x-ray liquidography

Jun Heo [1,2,§], Jong Goo Kim [1,2,§], Eun Hyuk Choi [1,2], Hosung Ki [1,2], Doo-Sik Ahn [1,2], Jungmin Kim[1,2], Seonggon Lee[1,2], Hyotcherl Ihee[1,2,]*

[1]Department of Chemistry and KI for the BioCentury, Korea Advanced Institute of Science and Technology (KAIST), Daejeon 34141, Republic of Korea

[2]Center for Advanced Reaction Dynamics, Institute for Basic Science (IBS), Daejeon 34141, Republic of Korea

[§]These authors contributed equally.

Corresponding Author

* hyotcherl.ihee@kaist.ac.kr (H.I)

Table of Contents

Supplementary Methods **Pages**

1. TRXL experiments ········ S4 - S5
2. Generation of time-resolved difference scattering curves ········ S6
3. Determining the molecular structure and atomic charge distribution of the ground state ········ S6 – S10
4. Singular value decomposition ········ S11
5. Structural analysis on ΔS_{iso} and ΔS_{aniso} (q, t > 300 fs)········ S12 – S17
6. Structural analysis on ΔS_{iso} and ΔS_{aniso} (q, $t \leq$ 300 fs) ········ S17 – S19
7. Anisotropic cage term ········ S20 – S24

Supplementary Notes **Pages**

1. Supplementary Note 1: The accuracy and limitations of the current analysis ······· S24 – S25

2. Supplementary Note 2: Simulations to quantify the sensitivities of isotropic and anisotropic signals on the shorter-bond and longer-bond dissociations ········ S26 – S27

References Pages

1. References ········ S50 – S51

Supplementary Figures

Pages

1. Supplementary Fig. 1 ········· S29
2. Supplementary Fig. 2 ········· S30
3. Supplementary Fig. 3 ········· S31
4. Supplementary Fig. 4 ········· S32
5. Supplementary Fig. 5 ········· S33
6. Supplementary Fig. 6 ········· S35
7. Supplementary Fig. 7 ········· S37
8. Supplementary Fig. 8 ········· S39
9. Supplementary Fig. 9 ········· S40
10. Supplementary Fig. 10 ········· S41
11. Supplementary Fig. 11 ········· S42
12. Supplementary Fig. 12 ········· S44
13. Supplementary Fig. 13 ········· S45
14. Supplementary Fig. 14 ········· S46
15. Supplementary Fig. 15 ········· S47
16. Supplementary Fig. 16 ········· S48
17. Supplementary Fig. 17 ········· S49

Supplementary Tables

Pages

1. Supplementary Table 1 ········· S28

Supplementary Methods

1. TRXL experiments

TRXL experiments were performed at the XSS beamline of PAL-XFEL (the Pohang Accelerator Laboratory x-ray free-electron laser). In the TRXL experiment, the sample solution of I_3^- was irradiated by an optical laser pulse to initiate a photoinduced reaction of the sample molecules, and a time-delayed x-ray pulse was used to probe the progress of the reaction. Femtosecond laser pulses at the center wavelength of 800 nm were generated from a Ti:sapphire regenerative amplifier and converted to 100-fs pulses at a wavelength of 400 nm by second-harmonic generation. The laser beam was focused by a lens to a spot of 260-μm diameter at the sample position, yielding a laser fluence of 1.8 mJ mm^{-2}. Femtosecond x-ray pulses were generated from the x-ray free-electron laser (XFEL) by self-amplified spontaneous emission. The x-ray pulses have center energy of 12.7 keV with a narrow energy bandwidth ($\Delta E/E$ = 0.3%). The x-ray beam was focused to a spot 40 μm in diameter at the sample position. The laser and x-ray beams were overlapped at the sample position with a crossing angle of 10°. The x-ray scattering patterns from the photoexcited I_3^- solution generated by the x-ray pulses were measured with an area detector (MX225-HS, Rayonix) over a q range of 1.0 $Å^{-1}$ to 7.0 $Å^{-1}$ with a sample-to-detector distance of 41 mm. The TRXL data were measured at various time delays in the range -0.6 ps to 6.9 ps with a time step of 0.1 ps, 7.2, 7.5, 7.8, 8.4, 8.7, 9.0, 9.3, 9.6, 12.2, 15.5, 19.6, 24.7, 31. 2, 39.4, 49.7, 62.7, 79.0, and 99.6 ps yielding a total of 95 time delays. Each scattering image was obtained with a single x-ray pulse and, to achieve a signal-to-noise ratio (SNR) sufficient for data analysis, around 200 images were acquired at each time delay. Especially for charge distribution analysis, an additional dataset covers 100 ps time delay with several reference time delays were consciously measured to achieve the high SNR, and around 2000 images were acquired for each time delay. The laser-off images were acquired with the x-ray pulse arriving 20 ps

earlier than the laser pulse (that is, with a −20 ps time delay) to probe the (unexcited) molecules in the ground state while ensuring the same average temperature of the sample solution. These laser-off images were repeatedly collected before every laser-on image and were subtracted from the laser-on images to yield time-resolved difference scattering patterns of the I_3^- solution. The resultant time-resolved difference scattering curves are shown in Supplementary Fig. 2. An I_3^- solution was prepared by mixing I_2 (from Aldrich, reagent grade) and KI (from Aldrich, reagent grade) with a 1:1 molar ratio in methanol with concentrations of 20 mM. In addition, 20 mM solution of 4-bromo-4′-(N,N-diethylamino)-azobenzene (HANCHEM, 99.9%) in methanol was prepared to measure the solvent heating signals. Since I_3^- ion is formed via equilibrium between (I_2 + I^-) and I_3^-, some of I_2 and I^- may still exist in solution. Those remaining reactants may cause complexity in the analysis of static x-ray scattering but not in the pump-probe scheme used in this work. Since I^- does not absorb at 400 nm and the extinction coefficient of I_2 at 400 nm is smaller than that of I_3^- by a factor of ~100, I_2 and I^- do not undergo photoinduced reactions effectively by 400 nm irradiation. Therefore, the scattering signals from the remaining reactants (I_2 and I^-) are canceled off by the pump-probe differencing scheme and do not appear in the difference scattering data. The sample solution was excited by the laser pulses of 400 nm wavelength. The sample solution was circulated through a nozzle with a 300-μm-thick aperture. To supply a fresh sample for every laser and x-ray shot, the flow velocity of the sample was set to be over 3 m s^{-1}. To prevent the scattering signal from contamination by radiation-damaged sample molecules, the sample in the reservoir was replaced with a fresh one whenever the transient signal measured at 100 ps was no longer reproduced. Even if the transient signal at 100 ps did not change, the sample in the reservoir was regularly replaced (every 2 ~ 3 h of measurement) to ensure the supply of fresh samples.

2. Generation of time-resolved difference scattering curves

We note that the two-dimensional scattering images have anisotropic components arising from the anisotropic orientational distributions of excited and ground-state molecules[1]. Two-dimensional scattering images recorded on the CCD detector were averaged and decomposed to give isotropic and anisotropic one-dimensional scattering curves, $S_{iso}(q,t)$ and $S_{aniso}(q,t)$ respectively, as a function of momentum transfer, q, and time delay, t, between the laser and x-ray pulses[2]. Time-resolved difference scattering curves, $\Delta S_{iso}(q,t)$ and $\Delta S_{aniso}(q,t)$, were generated by subtracting the reference data measured at –20 ps from the data at other time delays. Extracted $q\Delta S_{iso}(q,t)$ and $q\Delta S_{aniso}(q,t)$ are shown in Supplementary Fig. 2 in the form of contour plots and Supplementary Fig. 14 in the form of individual curves.

3. Determining the molecular structure and atomic charge distribution of the ground state

Since the structural dynamics from 100 ps to 3 μs were previously studied by TRXL[3], we first checked the data at 100 ps to ensure that the expected species were observed at 100 ps. As can be seen in Supplementary Fig. 3, the two scattering curves at 100 ps measured at the XFEL and synchrotron coincide with each other after adjusting the contributions of the solvent heating curve and correcting the effect arising from the different x-ray wavelength spectra. Noticeably, the 100-ps scattering curve measured at the XFEL has a significantly better signal-to-noise ratio (SNR) than the previously reported curve measured at the synchrotron[4]. Thanks to the better SNR achieved in the TRXL data presented in this work, we were able to further optimize the structure of I_3^- in the ground state, yielding the asymmetric bent structure shown in Fig. 2b.

To determine the structure and charge distribution of I_3^- in the ground state, a structural fitting analysis was performed using ΔS_{az} at 100 ps. According to previous studies

on the reaction dynamics of I_3^-, I_2^- and I radical are the only dominant species at 100 ps, and thus we considered Debye scattering curves of I_3^-, I_2^- and I radical for calculating the solute term in the structural fitting analysis (see Fig. 1b). The cage term was calculated with the aid of MD simulations, and the solvent term was experimentally acquired in a separate experiment with a heating dye (4-bromo-4′-(N,N-diethylamino)-azobenzene) in methanol solution (see Methods and Supplementary Fig. 5 for details). We note that only theoretical ΔS_{iso} were considered for the structural fitting analysis since the contribution of ΔS_{aniso} is negligible at 100 ps as the rotational dephasing processes are completed before 100 ps, as can be seen in Supplementary Fig. 2.

The structural fitting analysis was performed by optimizing structural parameters of I_3^- (bond lengths of I_A-I_B (R_{AB}) and I_B-I_C (R_{BC}), and I_A-I_B-I_C angle (θ)) and I_2^- (the I-I bond length ($R(I_2^-)$), where the three iodine atoms of I_3^- are labeled as I_A, I_B, and I_C, respectively, as depicted in Fig. 1a. The cage terms were prepared before the structural fitting analysis for specific molecular geometries and charge distributions. Here, the basic strategy of the structural fitting analysis is to perform the analysis iteratively until the solute structures to be optimized through the fitting process, and the structures used to precalculate the cage term become self-consistent. Initially, a cage term of I_3^- was calculated by an MD simulation performed against the symmetric linear structure (R_{AB} = R_{BC} = 2.95 Å, θ = 180°) and symmetric charge distribution ((I_A, I_B, I_C) = (-0.5 e, 0 e, -0.5 e)), which is the optimized values from a density functional theory (DFT) calculation on I_3^- (see the section "Density functional theory calculation" in Methods for the details on the DFT calculation). The cage term of I_2^- for the analysis was initially determined using the structure calculated from a DFT calculation ($R(I_2^-)$ = 3.23 Å). Throughout the entire analysis, we used an atomic charge of -0.5 e for two iodine atoms of I_2^- since it is evident that the negative charge is equally distributed to the two iodine atoms. Then, the structural fitting analysis was performed using

these cage terms by optimizing the structures of I_3^- and I_2^-. The structure of I_3^- resulting from the initial structural fitting has an asymmetric bent structure (R_{AB} = 3.14 ± 0.01 Å, R_{BC} = 2.92 ± 0.01 Å, θ = 151.2 ± 0.3°). The structure of I_2^- was determined to be R(I_2^-) = 3.28 ± 0.01 Å. These resulting structures of I_3^- and I_2^- are not self-consistent with the structure used for the calculation of the cage term. Moreover, the asymmetric bent structure of I_3^- raises the possibility that the one quantum of the negative charge is asymmetrically distributed over three iodine atoms, unlike the symmetric linear structure, where the charge should be symmetrically distributed.

Therefore, we pursued further refining the structure and atomic charge distribution of I_3^- based on the TRXL data. Before performing the structural fitting analysis again, we checked the sensitivity of the difference scattering curve on the structure and charge distribution of I_3^- by comparing cage terms calculated for several molecular geometries and charge distributions, as shown in Supplementary Fig. 4. Supplementary Fig. 4a-c, show simulated MD snapshots of I_3^- and solute-solvent pair distribution functions (PDFs) obtained for three representative combinations of different geometries and charge distributions. Supplementary Fig. 4a shows simulated results for the linear symmetric structure (R_{AB} = R_{BC} = 2.95 Å, θ = 180°) and the symmetric charge distribution ((I_A, I_B, I_C) = (-0.5 e, 0 e, -0.5 e)) of I_3^-, which is obtained from the DFT calculation. In the cases shown in Supplementary Fig. 4b, c, I_3^- have significantly different charge distributions, a symmetric charge distribution ((I_A, I_B, I_C) = (-0.5 e, 0 e, -0.5 e)) and an asymmetric charge distribution ((I_A, I_B, I_C) = (0 e, 0 e, -1.0 e)), respectively, with the asymmetric bent structure of I_3^- (R_{AB} = 3.14 Å, R_{BC} = 2.92 Å, θ = 151.2°) determined the previous structural fitting analysis. As can be seen from the PDFs for the three cases in Supplementary Fig. 4a-c, a PDF is more sensitive to the atomic charge distribution than to the molecular geometry of I_3^-. The cage terms calculated for the three cases are distinguishable from each other, as shown in Supplementary Fig. 4d. The simulation

results infer that the charge distribution affects both the solvent arrangement around the solute and accordingly alters the cage term in the TRXL signal, demonstrating the possibility of determining the charge distribution from the TRXL data based on the change of the cage term.

To consider the atomic charge distribution of I_3^-, new cage terms were generated by running MD simulations with the asymmetric bent I_3^- that have various charge distributions. Because MD simulations are slow processes, they could not be simply implemented into a maximum likelihood fitting procedure. To cope with this situation, we conducted grid search procedures by generating grid points of all possible atomic charge distributions. Generally, a typical driving force for uneven charge distribution comes from the difference in electronegativities. Since I_3^- is made of a single kind of atoms, three iodines, and the overall charge of I_3^- is -1, that is 1 e^-, it would be unlikely that any positive atomic charges are generated. Furthermore, previous theoretical studies using *ab initio* MD and CASSCF reported that no positive partial charge occurs on any iodine atom of I_3^- in various solvents unless the molecular geometry is significantly different from the equilibrium structure[5,6]. For that reason, we considered only negative atomic charges ranging from 0 to 1 e^-, so that we generated 66 grid points in total with a grid point representing a possible combination of three atomic charges as shown in Fig. 2a and Supplementary Fig. 5. For each grid point, which is a given charge distribution, we ran an MD simulation to obtain the corresponding cage term while the asymmetric bent structure of I_3^{--} determined from the initial structure fitting was used. Details on the MD simulations are described in Methods. Once all cage terms for all grid points were ready, we performed a fitting procedure for each grid point that refines the solute structures. Subsequently, we performed an additional optimization of the cage term and solute structures to attain self-consistency. Cage terms were calculated again using the refined structures of I_3^- and I_2^- determined in the previous fitting procedure for all

the grid points. Finally, using the refined cage terms, the solute structures were further optimized via structural fittings for the grid points. The detailed procedure of the grid search is depicted in Supplementary Fig. 5. A plot of the weighted R-factors (wR) for all grid points (that is, a grid map) shown in Fig. 2a visualizes which grid point has the minimum wR[7,8]. According to the grid search results, the charge distribution of (I_A, I_B, I_C) = (-0.9 e, 0.0 e, -0.1 e) with an asymmetric bent structure (R_{AB} = 3.09 ± 0.01 Å, R_{BC} = 2.96 ± 0.01 Å and θ = 152 ± 0.4°) yielded the smallest wR. More details of the resulting fitting parameters are summarized in Supplementary Table 1. The optimized atomic charge distribution indicates that the negative charge is asymmetrically located in I_3^-. Specifically, most of the negative charge is localized on the terminal I atom (I_A) forming the longer I-I bond (I_A-I_B) in the ground state. We further inspected how the TRXL signal is altered depending on atomic charge distributions by comparing the fitting qualities at three representative grid points where most of the negative charge is localized on (i) I_A or (ii) I_B or (iii) equally distributed to I_A and I_C, as shown in Fig. 2c. The comparison reveals that the various charge distributions are distinguishable from the TRXL data and the different charge distributions predominantly affect the TRXL signal in a small-angle region (q < 3 Å^{-1}), demonstrating that the employed analysis for calculating cage terms considering both the molecular geometry and charge distribution has provided the quantitative determination of the atomic charge distribution. We also checked the possibility of overfitting and model bias by removing randomly chosen 10% of the data points, fitting only the remaining 90% of the data, and checking the weighted R-free factors (wR_{free}). The wR_{free} (0.0683) is not significantly larger than the wR (0.0681), indicating that the obtained fit result did not suffer from overfitting or bias. Moreover, another evidence supporting this finding is provided by considering the rotational dynamics, which turn out to depend on the charge distribution (Fig. 2d), as discussed in the main text.

4. Singular value decomposition

To investigate the time-dependent features in the experimental data of I_3^-, we applied the singular value decomposition (SVD) analysis to the measured isotropic and anisotropic TRXL data. For the SVD analysis, we built an $\mathrm{n}_q \times \mathrm{n}_t$ data matrix, $\mathbf{A}$, whose column vectors are experimental time-resolved difference scattering curves, where n_q is the number of q points in the difference scattering curves and n_t is the number of time-delay points. As a result of SVD, the matrix $\mathbf{A}$ is decomposed into three matrices satisfying the relationship of $\mathbf{A} = \mathbf{USV}^{\mathrm{T}}$. $\mathbf{U}$ is an $\mathrm{n}_q \times \mathrm{n}_t$ matrix whose column vectors are called left singular vectors (lSVs) of $\mathbf{A}$, $\mathbf{V}$ is an $\mathrm{n}_t \times \mathrm{n}_t$ matrix whose column vectors are called right singular vectors (rSVs) of $\mathbf{A}$, and $\mathbf{S}$ is a diagonal $\mathrm{n}_t \times \mathrm{n}_t$ matrix whose diagonal elements are called singular values of $\mathbf{A}$. The matrices $\mathbf{U}$ and $\mathbf{V}$ follow the relationships of $\mathbf{U}^{\mathrm{T}}\mathbf{U} = \mathbf{I}_{\boldsymbol{n_t}}$ and $\mathbf{V}^{\mathrm{T}}\mathbf{V} = \mathbf{I}_{\boldsymbol{n_t}}$, respectively, where $\mathbf{I}_{\boldsymbol{n_t}}$ is an identity matrix. The lSVs represent time-independent q-spectra, the rSVs represent time-dependent amplitude changes of corresponding lSVs, and the singular values represent the weights of the corresponding lSVs and rSVs. Since the singular values are ordered so that $\mathrm{s}_1 \geq \mathrm{s}_2 \geq \cdots \geq \mathrm{s}_n \geq 0$, (both left and right) singular vectors on more left are supposed to have larger contributions to the experimental data matrix $\mathbf{A}$. The results of SVD on of the isotropic and anisotropic $q\Delta S(q,t)$ are summarized in Supplementary Fig. 12. We used the SVD results to construct the proper fitting model for the scattering data. Because SVD extracts the feature and time-dependent character of the scattering components, we can quantify the main contributors to the dynamics and the corresponding time constants. Also, we applied SVD to the anisotropic heating signal to obtain the contribution of the Kerr effect of solvent molecules, which was used for the structural analysis. More details are described in the section “5. Structural analysis on ΔS_{iso} and ΔS_{aniso} (q, t > 300 fs)”.

5. Structural analysis on ΔS_{iso} and ΔS_{aniso} (q, t > 300 fs)

The difference scattering curves can be described by a sum of three terms: (i) the solute term, (ii) the solvent term, and (iii) the cage term (solute-solvent cross term)[9]. For the structural fitting analysis, we adjusted several parameters related to those contributing terms in TRXL data by minimizing the discrepancy between the theoretical and experimental data. To quantify the agreement between the calculated and experimental difference scattering curves, we calculated the weighted R-factor (wR) using the following equation.

$$\mathrm{wR}(t) = \sqrt{\frac{\sum_i \frac{\left(\Delta S_{theory,iso}(q_i,t) - \Delta S_{\mathrm{iso}}(q_i,t)\right)^2}{(\sigma_{\mathrm{iso}}(q_i,t))^2} + \sum_i \frac{\left(\Delta S_{theory,aniso}(q_i,t) - \Delta S_{\mathrm{aniso}}(q_i,t)\right)^2}{(\sigma_{\mathrm{aniso}}(q_i,t))^2}}{\sum_i \frac{\left(\Delta S_{\mathrm{iso}}(q_i,t)\right)^2}{(\sigma_{\mathrm{iso}}(q_i,t))^2} + \sum_i \frac{\left(\Delta S_{\mathrm{aniso}}(q_i,t)\right)^2}{(\sigma_{\mathrm{aniso}}(q_i,t))^2}}} \quad \text{(S1)}$$

In the equation, ΔS_{iso} and ΔS_{aniso} stand for the experimentally measured isotropic and anisotropic difference scattering signals. $\Delta S_{theory,iso}$ and $\Delta S_{theory,aniso}$ are the theoretical difference scattering curves calculated during the fitting processes, and σ_{iso} and σ_{aniso} are the standard errors of ΔS_{iso} and ΔS_{aniso}, respectively. The minimization of the wR was performed to refine the molecular structure using the MINUIT package written at CERN and the error analysis was performed by MINOS, a built-in algorithm in the MINUIT software[10]. Structural and anisotropic parameters were optimized by minimizing the wR. The structure of ground state I_3^- was fixed to that determined from the analysis of the 100-ps data. The fitting process was conducted for each time delay by constructing the theoretical difference scattering curves calculated using the following equations.

$$\Delta S_{theory,iso}(q,t) = B_0(t) * \left(S_{e,iso}(q,t) - S_{g,iso}(q,t)\right) + B_1(t) * \left.\frac{\partial S(q)}{\partial T}\right|_\rho \quad \text{(S2)}$$

$$\Delta S_{theory,aniso}(q,t) = B_0(t) * \left(A_e(t) * S_{e,aniso}(q,t) - A_g(t) * S_{g,aniso}(q,t)\right) + B_2(t) * \Delta S_{Kerr}$$

(S3)

$S_{e,iso}$ and $S_{g,iso}$ are the theoretical isotropic scattering curves of the products (I_2^- and I) and the reactant (I_3^-), respectively. $S_{e,aniso}$ and $S_{g,aniso}$ are the theoretical anisotropic scattering curves of I_2^- and I_3^-. These scattering curves, $S_{e,iso}$, $S_{g,iso}$, $S_{e,aniso}$, and $S_{g,aniso}$ are composed of the solute term and the cage term. $\left.\frac{\partial S(q)}{\partial T}\right|_\rho$ is the experimentally measured temperature solvent differential using a heating dye[11-13]. To obtain $\left.\frac{\partial S(q)}{\partial T}\right|_\rho$ and ΔS_{Kerr}, we measured TRXL data of a heating dye as described in the section "1. TRXL experiments" and extracted isotropic and anisotropic heating signals ($\Delta S_{heat,iso}$ and $\Delta S_{heat,aniso}$) as shown in Supplementary Figs. 13a and 13b, respectively. The isotropic heating signal at 100 ps shown in Supplementary Fig. 13c was used as $\left.\frac{\partial S(q)}{\partial T}\right|_\rho$. ΔS_{Kerr} is the anisotropic signal that originates from the Kerr effect of solvent molecules. To obtain ΔS_{Kerr}, we performed SVD analysis on the anisotropic heating signal ($\Delta S_{heat,aniso}$) shown in Supplementary Fig. 13b, and the 1st lSV shown in Supplementary Fig. 13d was used as ΔS_{Kerr} (The procedures for SVD analysis are detailed in the section "4. Singular value decomposition"). These experimentally measured signal using the heating dye, $\left.\frac{\partial S(q)}{\partial T}\right|_\rho$ and ΔS_{Kerr}, are shown in Supplementary Fig. 13. $B_0(t)$, $B_1(t)$ and $B_2(t)$ reflect the population dynamics of I_2^-, and the intensity of the solvent heating signal, and the intensity of the solvent Kerr signal, respectively. $A_e(t)$ and $A_g(t)$ are the anisotropy coefficients of I_2^- and I_3^-, respectively, and describe the anisotropy change of each molecule. Especially A_g, which shows anisotropy change of I_3^-, represents the anisotropy originating from the hole of depleted I_3^-. The fitted results are presented in Figs. 4 and 5. The isotropic scattering curves were calculated with the Debye equation[9], and the anisotropic scattering curves were calculated under the assumption of single-photon

excitation by which the anisotropic distribution of the excited molecules has $\cos^2\phi$ distribution along with the pump beam polarization[2].

To calculate $S_{e,iso}$ and $S_{g,iso}$ in Eq. (S2), the theoretical isotropic scattering curves, S_{iso}, were calculated as follows:

$$S_{iso}(q,t) = \sum_n \sum_m f_n(q) f_m(q) \frac{\sin q r_{nm}(t)}{q r_{nm}(t)} + S_{iso,cage} \quad \text{(S4)}$$

, where f_n and f_m are the x-ray form factors of atoms n and m in the molecule, r_{nm} is the interatomic distance between atoms n and m, and $S_{iso,cage}$ is the isotropic cage term, which can be calculated from the MD simulation. The theoretical isotropic scattering curves were calculated for the products (I_2^- and I) and the reactant (I_3^-) using Eq. (S4), yielding $S_{e,iso}$ and $S_{g,iso}$, respectively.

To calculate $S_{e,aniso}$ and $S_{g,aniso}$ in Eq. (S3), the theoretical anisotropic scattering curves, S_{aniso}, were calculated as follows:

$$S_{aniso}(q,t) = -\sum_n \sum_m f_n(q) f_m(q) P_2[\cos \xi_{nm}(t)] j_2[q r_{nm}(t)] + S_{aniso,cage} \quad \text{(S5)}$$

, where P_2 is the second-order Legendre polynomial, ξ_{nm} is the angle between r_{nm} and the laser polarization axis, j_2 is the second-order Bessel function, and $S_{aniso,cage}$ is the anisotropic cage term, which was obtained following the procedures detailed in the section "7. Anisotropic cage term". The theoretical anisotropic scattering curves were calculated for the products (I_2^- and I) and the reactant (I_3^-) using Eq. (S5), yielding $S_{e,aniso}$ and $S_{g,aniso}$, respectively.

In the fitting analysis, we constructed the theoretical isotropic and anisotropic scattering curves following Eqs. (S2) – (S5), and minimized the wR given by Eq. (S1), to achieve satisfactory fits to the experimental data. For the fitting, we optimized several structural parameters such as the I-I bond length ($R(I_2^-)$), the distance between the I radical and the center of I_2^- ($r(I\text{-}I^{2-})$), the root-mean-square displacement of the Debye-Waller factor

(DWF) for r(I-I_2^-) (σ) were used as fitting parameters. Here, we introduced the DWF (exp(-$\sigma^2 q^2/2$)) to describe the relatively free movement of the escaping I radical, yielding a broad distribution of the interatomic distance between the I radical and the center of I_2^-. We note that for a typical analysis of TRXL data, it is sufficient to use DWF of 1 (that is, σ = 0) unless an interatomic distance has an unusually broad distribution. When including the DWF, the theoretical scattering curves in Eqs. (S4) and (S5) are modified as follows:

$$S_{iso}(q,t) = \sum_n \sum_m f_n(q) f_m(q) \frac{\sin qr_{nm}(t)}{qr_{nm}(t)} e^{\left(\frac{-\sigma_{nm}^2 q^2}{2}\right)} + S_{iso,cage} \quad \text{(S6)}$$

$$S_{aniso}(q,t) = -\sum_n \sum_m f_n(q) f_m(q) P_2[\cos \xi_{nm}(t)] j_2[qr_{nm}(t)] e^{\left(\frac{-\sigma_{nm}^2 q^2}{2}\right)} + S_{aniso,cage} \quad \text{(S7)}$$

In summary, for the structural analysis on ΔS_{iso} and ΔS_{aniso} (q, t > 300 fs), R(I_2^-), r(I-I_2^-), σ, $A_e(t)$, $A_g(t)$, $B_0(t)$, $B_1(t)$ and $B_2(t)$ were used as the time-dependent fitting parameters. Among these parameters, R(I_2^-), r(I-I_2^-) and $B_0(t)$ were used for both isotropic and anisotropic fitting processes, σ and $B_1(t)$ were used for the isotropic curve fitting, and the others, $A_e(t)$, $A_g(t)$, and $B_2(t)$ were used to fit the anisotropic data.

Because there is no anisotropic signal at 100 ps, anisotropic terms are not considered for the refinement of the charge distribution of I_3^- and structure of I_2^- and I_3^-. For the refinement process of the 100-ps data, we also randomly chose 10% of the overall data and used the other 90% for the fitting process. Then the wR for the 10% data was used to calculate the weighted R-free factor (wR_{free}), whereas 90% of the data the wR. The wR_{free} was compared with the corresponding wR to check for any overfitting or bias.

We note that we used a static cage term, which was calculated from the cage structure that is obtained from the equilibrium MD simulation of the solute molecule having fixed molecular geometry and charge distribution, for each chemical species of I_3^-, I_2^- and I. An example for $I_3^- \rightarrow I_2^- + I$ is shown in Supplementary Fig. 16. In fact, it is known that the cage structure around a solute during a reaction is not static but dynamic. There are two

dynamic aspects to be considered. The first is that the structure of solute, for example, I_2^-, is not static but dynamic: at early time delays, I_2^- adapts non-equilibrium structure, which is slowly relaxed to the equilibrium structure along with the vibrational relaxation. In this process, the cage structure around I_2^- may dynamically adapt to the structural relaxation of I_2^-. For example, the cage structure for the vibrationally excited I_2^- ($R(I_2^-)$ = 3.34 Å at t = 0.7 ps) would in principle be different from that for the I_2^- in equilibrium ($R(I_2^-)$ = 3.25 Å). Nevertheless, according to our simulation, as shown in Supplementary Fig. 16, such an adaptation of the cage structure around the relaxing I_2^- molecule does not have a significant contribution to the TRXL signal, indicating that such a dynamic cage structure around I_2^- is negligible in our analysis.

In addition to this, the potential contribution of solvation dynamics, the non-equilibrium cage structure, should also be taken into account. To check whether the contribution of the solvation dynamics is evident in our TRXL data, we compared two different models, (i) a non-equilibrium cage model with the assumption that, at the earliest time delays, the cage has not yet fully adapted to the rapid change in molecular structures and charge distribution accompanying the reaction $I_3^- \rightarrow I_2^- + I$ so that the structure of the cage would be much more similar to that of the parent molecule, I_3^-, than that of the products, I_2^- and I, and (ii) an equilibrium cage model with the assumption that, even at the earliest time delays, the cage has already been fully adapted to the rapid change so that the structure of the cage is similar to that of the products, I_2^- and I. While the former model assumes that the solvation dynamics is relatively slow so that it can be captured with the IRF of the current TRXL experiment, the latter model assumes that the solvation dynamics is too fast to be captured with the IRF of the current experiment. For the former model, the cage term was calculated with an approximation that in the non-equilibrium cage structure, only the structure of the solute molecule is changed while the positions and orientations of

surrounding solvent molecules are retained as the same as those in the equilibrium ground state. Following this approximation, the non-equilibrium cage structure was obtained simply by replacing I_3^- in the equilibrium MD snapshots of I_3^- with the solute molecule in the excited state while retaining the surrounding solvent molecules. Comparison of the goodness of fit for the two different cage models shows that the quality of the fit is not better, and even worse, for the non-equilibrium cage model (see Supplementary Fig. 17). It was reported from literature that the solvation dynamics of dye molecules in methanol shows three time constants: ~100 fs, 1.1 ps, and 10 ps[14]. We suggest that the current TRXL data could not capture the signal corresponding to the solvation dynamics probably because most of the structural relaxation of the cage is finished with the 100 fs time constant, which is too fast to be captured with the IRF of our experiment, 300 fs. Also, the solvation dynamics associated with the other two time constants were not observed in our TRXL data, implying that the structural change occurring with the two time constants of 1.1 ps and 10 ps would be much smaller. Accordingly, we used the time-independent, equilibrium cage model to describe the cage structure of solute molecules throughout the fitting process.

6. Structural analysis on ΔS_{iso} and ΔS_{aniso} (q, $t \leq 300$ fs)

For the structural analysis on the experimental curves within the experimental IRF ($t \leq 300$ fs), we considered the convolution of the molecular response with the IRF instead of performing the structural analysis on the individual experimental curves. Thus, an approach to fit the experimental curves within the experimental IRF ($t \leq 300$ fs) was slightly modified from that used for the time delays larger than the experimental IRF, following the previously reported procedures[15]. The time-dependent parameters for the structures of I_2^- and the dissociating I radical, σ of the DWF, anisotropy coefficients ($A_e(t)$, and $A_g(t)$) and the population of I_2^- ($B_0(t)$) were modeled by a quartic polynomial function.

$$x(t) = \sum_{k=0}^{4} a_{4-k} t^{4-k} \quad \text{(S8)}$$

, where $a_{4\text{-}k}$ is the coefficient of the polynomial function. The isotropic and anisotropic scattering curves for the solute signal in $S_{inst,iso}(q, t)$, $S_{inst,aniso}(q, t)$ were calculated based on $x(t)$ from which the molecular structures were constructed and the DWF for the dissociating I_3^- was calculated. Subsequently, the instantaneous theoretical difference scattering curves, $\Delta S_{inst}(q, t)$, were calculated using the following equations:

$$\Delta S_{inst,iso}(q,t) = B_0(t) * \left[S_{e,iso}(q,t) - S_{g,iso}(q)\right] + B_1(t) * \left.\frac{\partial S(q)}{\partial T}\right|_{\rho} \quad \text{(S9)}$$

$$\Delta S_{inst,aniso}(q,t) = B_0(t) * \left[S_{e,aniso}(q,t)A_e(t) - S_{g,aniso}(q)A_g(t)\right] + B_2(t) * \Delta S_{Kerr} \quad \text{(S10)}$$

, where the terms in Eqs. (S9) and (S10) are the same as those in Eqs. (S2) and (S3). The fractions of the heating ($B_1(t)$) and Kerr ($B_2(t)$) signals were determined with the SANOD during the fitting process minimizing the residual of the fit[16]. $B_0(t)$, $A_e(t)$, and $A_g(t)$ were also modeled using quadratic polynomial functions shown in Eq. (S8) and the coefficients of the polynomial functions were used as fitting parameters. Then, $\Delta S_{inst,iso}(q, t)$ and $\Delta S_{inst,aniso}(q, t)$ were convoluted by the experimental IRF, IRF(t), with a full width at half-maximum (FWHM) of 270 fs. The experimental IRF was determined from the fitting of rSVs (Supplementary Fig. 12). The convolution resulted in $\Delta S_{theory,iso}(q, t \leq 300$ fs) and $\Delta S_{theory,aniso}(q, t \leq 300$ fs) as shown in the following equations.

$$\Delta S_{theory,iso}(q, t \leq 300\, fs) = \Delta S_{inst,iso}(q,t) \otimes IRF(t) \quad \text{(S11)}$$

$$\Delta S_{theory,aniso}(q, t \leq 300\, fs) = \Delta S_{inst,aniso}(q,t) \otimes IRF(t) \quad \text{(S12)}$$

Under the constraints where the polynomial functions smoothly connect the structure at 0 fs, which is the structure of the I_2^- at Frank-Condon region, and the structures at 300 fs determined following the procedures described in "Structural analysis on ΔS_{iso} and ΔS_{aniso} (q, t > 300 fs)" of Supplementary Information, the coefficients of the polynomial functions for each structural parameter were optimized by minimizing the wR using the following equation:

$$wR = \sqrt{\frac{\sum_{j=time\ delay}\sum_i \left[\frac{\left(\Delta S_{theory,iso}(q_i,t_j) - \Delta S_{\text{iso}}(q_i,t_j)\right)^2}{\left(\sigma_{\text{iso}}(q_i,t_j)\right)^2} + \frac{\left(\Delta S_{theory,aniso}(q_i,t_j) - \Delta S_{\text{aniso}}(q_i,t_j)\right)^2}{\left(\sigma_{\text{aniso}}(q_i,t_j)\right)^2}\right]}{\sum_{j=time\ delay}\sum_i \left[\frac{\left(\Delta S_{\text{iso}}(q_i,t_j)\right)^2}{\left(\sigma_{\text{iso}}(q_i,t_j)\right)^2} + \frac{\left(\Delta S_{\text{aniso}}(q_i,t_j)\right)^2}{\left(\sigma_{\text{aniso}}(q_i,t_j)\right)^2}\right]}} \quad \text{(S13)}$$

, where σ_{iso} and σ_{aniso} is the standard deviation of the isotropic and anisotropic difference scattering intensity at each q and t. Finally, the resultant $\Delta S_{theory,iso}(q, t \leq 300$ fs) and $\Delta S_{theory,aniso}(q, t \leq 300$ fs) were concatenated with $\Delta S_{theory,iso}(q, t > 300$ fs) and $\Delta S_{theory,aniso}(q, t > 300$ fs), respectably, giving rise to $\Delta S_{theory,iso}$ and $\Delta S_{theory,aniso}$ shown in Figs. 4a and 5a. The optimized $x(t)$ values were concatenated with the corresponding parameters used to calculate the solute signal of $\Delta S_{theory,iso}(q, t > 300$ fs) and $\Delta S_{theory,aniso}(q, t > 300$ fs) and are represented in Figs. 4b, 4c, 5b and 5c. The standard deviations for the structural parameters and σ of the DWF at $t \leq 300$ fs were calculated based on the covariance matrix, which was calculated during the fit of the coefficients of quartic polynomials using the following equation:

$$\sigma_{x(t)} = \sqrt{\sum_{k=0}^{4}(t^{4-k})^2 Var(a_{4-k}) + 2\sum_{i=0}^{4}\sum_{j=i+1}^{4} t^{4-i}t^{4-j}Cov(a_{4-i}, a_{4-j})}$$

(S14)

, where $\sigma_{x(t)}$ is the standard deviation of the parameter x at time delay t, $Var(a_{4-k})$ is the variance of the coefficient a_{4-k}, and $Cov(a_{4-i}, a_{4-j})$ is the covariance between the coefficients a_{4-i} and a_{4-j}.

In summary, for the structural analysis on ΔS_{iso} and ΔS_{aniso} (q, $t \leq 300$ fs), $R(I_2^-)$, $r(I-I_2^-)$, σ, $A_e(t)$, $A_g(t)$, and $B_0(t)$ were used as the time-dependent fitting parameters. Among these parameters, $R(I_2^-)$, $r(I-I_2^-)$ and $B_0(t)$ were used for both isotropic and anisotropic fitting processes, σ was used for the isotropic curve fitting, and the others, $A_e(t)$, and $A_g(t)$ were used to fit the anisotropic data.

7. Anisotropic cage term

The change of solute structure is accompanied by the change of cage structure around the solute molecule. The cage term for the randomly oriented case can be calculated using the pair distribution functions (PDFs) for the cross pairs of an atom of a solute molecule and an atom of the solvent molecule. If solute molecules are isotropically oriented, the cage term can be calculated with the following isotropic scattering equation[9]:

$$S_{cage}(q) = 2 * \sum_{j}\sum_{k} f_j(q)f_k(q)\, n_k \int 4\pi r^2 (g_{jk}(r) - 1)\frac{\sin qr}{qr} dr$$

(S15)

, where j is the index of an atom in the solute molecule; k is the index of an atom in the solvent molecule; n_k stands for the number density of atom k, and g_{jk} means the PDF of the atomic pair j and k; q is the absolute value of the momentum transfer vector; and r is the distance between atom j and atom k; $f_j(q)$ and $f_k(q)$ are the atomic form factor of atom j and atom k for q. Eq. (S15) cannot be used to calculate the anisotropic cage term, which requires the consideration for the anisotropic environment around the solute molecule. We have developed a methodology to calculate the anisotropic cage term for any desired distribution of the solute molecules, as explained in the following. First, we will explain it for the scattering from a single solute molecule embedded in solvent molecules and then expand it to the case with the ensemble with angular distribution induced by the photoselection. Eq. (S15) is not adequate for the calculation of any anisotropic term because $g_{jk}(r)$ in Eq. (S15) does not contain orientational information around a solute molecule. Therefore, we started from the following generalized equation from which Eq. (S15) is derived for the special case of randomly oriented molecules[17]:

$$S(\vec{\boldsymbol{q}}) = \sum_n \sum_{n'} f_n(|\vec{\boldsymbol{q}}|) f_{n'}(|\vec{\boldsymbol{q}}|)\ cos(\vec{\boldsymbol{q}} \cdot \overrightarrow{\boldsymbol{r}_{nn'}}) \quad \text{(S16)}$$

, where $\vec{\boldsymbol{q}}$ is the momentum transfer vector, and $\overrightarrow{\boldsymbol{r}_{nn'}}$ stands for the displacement vector

between atom n and atom n'. From this equation, we can construct the following generalized equation for calculating the cage term applicable to arbitrarily oriented cases:

$$S_{cage}(\vec{\boldsymbol{q}}) = 2 * \sum_{j}\sum_{k} f_j(|\vec{\boldsymbol{q}}|) f_k(|\vec{\boldsymbol{q}}|)\, n_k \iiint_{r,\phi,\theta} r^2\big(g_{jk}(r,\phi,\theta) - 1\big)\ cos(\vec{\boldsymbol{q}} \cdot \vec{\boldsymbol{r}})\ \ dr\, d\phi\, d\theta$$

(S17)

, where $r, \phi,$ and θ are the spherical coordinates of the displacement vector, $\vec{\boldsymbol{r}}$, and stand for a radial distance, a polar angle, and an azimuthal angle, respectively; $g_{jk}(r,\phi,\theta)$ is the spatial distribution function (SDF). Note that a PDF is a function of *r*, and an SDF is a function of $\vec{\boldsymbol{r}}$. One drawback with Eq. (S17) is that it requires the calculation of $g_{jk}(r,\phi,\theta)$, which takes too much workforce. For this reason, we further modified Eq. (S17) by replacing $g_{jk}(r,\phi,\theta)$ with an orientation-dependent PDF, $g_{jk}(r,\overrightarrow{\boldsymbol{v_m}})$ so that the cage term from explicit solute and solvent molecules in an MD snapshot can be easily calculated, as detailed below. First, we defined unit angular vectors ($\overrightarrow{\boldsymbol{v_m}}$), which point evenly spaced spherical coordinates on the unit sphere using the spiral methods[18]. This unit angular vectors can be written as,

$$\theta_m = \sqrt{\pi N}\sin^{-1}(\tfrac{2m-1-N}{N}) \quad \text{(S18-1)}$$

$$\phi_m = \cos^{-1}(\tfrac{2m-1-N}{N}) \quad \text{(S18-2)}$$

$$\overrightarrow{\boldsymbol{v_m}} = [\cos\theta_m \sin\phi_m , \sin\theta_m \sin\phi_m , \cos\phi_m] \quad \text{(S18-3)}$$

, where N is the total number of unit angular vectors, and m = 1,2, …, *N*, and θ_m and ϕ_m are the azimuthal and polar angles of the m-th unit angular vector, respectively. The *N* of 1,500 was used for the calculation. Each dot in Supplementary Fig. 6a represents each unit angular vector that spans the surface of the unit sphere in 3D space. A unit angular vector indicates the direction of the corresponding orientation-dependent PDF to construct, $g_{jk}(r,\overrightarrow{\boldsymbol{v_m}})$. For constructing $g_{jk}(r,\overrightarrow{\boldsymbol{v_m}})$, we first expanded the unit sphere so that the expanded sphere can

contain the whole MD box used for an MD simulation. An expanded sphere with a radius of 20 Å satisfied this condition (Supplementary Fig. 7a). Then, the expanded sphere around the solute molecule is divided into polyhedral cones with uniform solid angles so that each polyhedral cone has a height of 20 Å, as illustrated in Supplementary Fig. 6b. To calculate the orientation-dependent PDF, $g_{jk}(r, \overrightarrow{\boldsymbol{v_m}})$, we identified the desired atoms (for example, *k*) of solvent molecules within the polyhedral cone of the expanded sphere and calculated the PDF for the cross pair of an atom of a solute molecule (for example, *j*) and the selected solvent atoms within each polyhedral cone specified by $\overrightarrow{\boldsymbol{v_m}}$. The cage signal corresponding to the orientation-dependent PDF is given by the following equation.

$$S_{cage}(\vec{\boldsymbol{q}}, \overrightarrow{\boldsymbol{v_m}}) = 2 * \sum_j \sum_k f_j(|\vec{\boldsymbol{q}}|) f_k(|\vec{\boldsymbol{q}}|) n_k \int \frac{4\pi r^2}{N} (g_{jk}(r, \overrightarrow{\boldsymbol{v_m}}) - 1) \, cos(r\vec{\boldsymbol{q}} \cdot \overrightarrow{\boldsymbol{v_m}}) \, dr$$

(S19)

Then, the cage signal for a single solute molecule embedded in solvent molecules is calculated by summing all $S_{cage}(\vec{\boldsymbol{q}}, \overrightarrow{\boldsymbol{v_m}})$, as follows.

$$S_{cage}(\vec{\boldsymbol{q}}) = \sum_{m=1}^{N} S_{cage}(\vec{\boldsymbol{q}}, \overrightarrow{\boldsymbol{v_m}})$$

$$= 2 * \sum_m^N \sum_j \sum_k f_j(|\vec{\boldsymbol{q}}|) f_k(|\vec{\boldsymbol{q}}|) n_k \int \frac{4\pi r^2}{N} (g_{jk}(r, \overrightarrow{\boldsymbol{v_m}}) - 1) \, cos(r\vec{\boldsymbol{q}} \cdot \overrightarrow{\boldsymbol{v_m}}) \, dr$$

(S20)

The resulting Eq. (S20) is equivalent to Eq. (S17), except that it is modified by decomposing the SDF, $g_{jk}(r, \phi, \theta)$, to orientation-dependent PDFs, $g_{jk}(r, \overrightarrow{\boldsymbol{v_m}})$, to reduce the complicity that arises from calculating the SDF. Finally, using Eq. (S20), it is possible to calculate the scattering pattern from the solute-solvent pair for a solute molecule with any orientation. Generally, a single MD snapshot cannot give a satisfactory cage signal due to the insufficient sampling of the positions and orientations of solvent molecules. For this reason, a

sufficient number of MD snapshots need to be generated from MD simulations, and the calculated $S_{cage}(\vec{\boldsymbol{q}}, \overrightarrow{\boldsymbol{v_m}})$ from those MD snapshots need to be averaged. For example, if 200,000 snapshots from the MD simulation are extracted, then the orientation-dependent PDFs calculated from those MD snapshots are averaged for each unit angular vector as follows: (Supplementary Fig. 7c).

$$g_{jk}^{ave}(r, \overrightarrow{\boldsymbol{v_m}}) = \sum_{i=1}^{200,000} g_{jk}^{(i)}(r, \overrightarrow{\boldsymbol{v_m}})$$

(S21)

, where $g_{jk}^{(i)}(r, \overrightarrow{\boldsymbol{v_m}})$ is the orientation-dependent PDF calculated from the *i*-th MD snapshot and $g_{jk}^{ave}(r, \overrightarrow{\boldsymbol{v_m}})$ is the averaged orientation-dependent PDFs over 200,000 MD snapshots. Using the averaged orientation-dependent PDFs ($g_{jk}^{ave}(r, \overrightarrow{\boldsymbol{v_m}})$), the cage scattering pattern arising from the anisotropic orientation of the solute molecules is calculated with the following equation.

$$S_{cage}'(\vec{\boldsymbol{q}}) = \sum_{m}^{N} \sum_{j} \sum_{k} f_j(|\vec{\boldsymbol{q}}|) f_k(|\vec{\boldsymbol{q}}|) n_k \int \frac{4\pi r^2}{N} (g_{jk}^{ave}(r, \overrightarrow{\boldsymbol{v_m}}) - 1) \, cos(r\vec{\boldsymbol{q}} \cdot \overrightarrow{\boldsymbol{v_m}}) \, dr$$

(S22)

So far, we explained how the cage scattering term for a single solute molecule with a certain fixed orientation embedded in a box of solvent molecules.

The next step is to consider the effect of the anisotropic distribution induced by the photoselective alignment. The single-photon excitation process induced by a linearly polarized laser pulse generates an anisotropic distribution of excited molecules and the surrounding solvents, which initially has the cosine-squared distribution[2]. Then, the anisotropic distribution of excited molecules is given by $P(\phi) \propto \cos^2\phi$, where ϕ stands for the angle between the laser polarization and the transition dipole moment. To consider the

effect of molecular orientations, we rotated each MD snapshot to retrieve the molecular orientations induced by the photoexcitation where the rotated solute molecules have the $\cos^2$ distribution, as shown in Supplementary Fig. 7b. For each of the rotated MD snapshots, the corresponding orientation-dependent PDFs were calculated following the procedures described above. Then, the orientation-dependent PDFs calculated from the rotated MD snapshots were averaged for each unit angular vector using Eq. (S21). Using the averaged orientation-dependent PDFs ($g_{jk}^{ave}(r, \overrightarrow{\boldsymbol{v_m}})$), the cage scattering pattern arising from the anisotropic orientation of the solute molecules was calculated with Eq. (S22). Finally, isotropic and anisotropic cage terms were extracted from $S_{cage}'(\vec{\boldsymbol{q}})$ following the previously reported procedures[1] as shown in Supplementary Fig. 8.

Supplementary Notes

Supplementary Note 1: The accuracy and limitations of the current analysis

In the current work, classical MD simulations, which do not treat hydrogen bonding explicitly, were used to account for the distribution of the solvent molecules around I_3^- whose structure is frozen during the simulation. Due to the possibility of interdependence among the geometry, the charge distribution, and the hydrogen bonding of I_3^-, a more sophisticated *ab initio* MD simulation taking these into account more explicitly may provide a more accurate result than the classical MD simulation employed in the current work. Nevertheless, it should be noted that the molecular structure of I_3^- obtained from the currently available *ab initio* MD simulation itself shows difference from the experimentally determined structure yet. More specifically, whereas the two I-I distances of the reported average structure from an *ab initio* MD simulation (2.94 Å and 3.09 Å) agree well with those (2.96 Å and 3.09 Å) of the optimized structure from the TRXL data, the I-I-I angle from the *ab initio* MD simulation (170.6°) is noticeably larger than that from the TRXL data (152.0°). For this reason, we

optimized both the molecular structure and charge distribution based on the TRXL data. The structural fitting of the molecular structure partly compensates the current imperfection of *ab initio* MD simulations. For example, regarding the interdependence of the structure and charge distribution, the molecular structure is mostly determined by the high q data where the influence of the cage term is small. Therefore, the molecular structure can be accurately determined from the high q region of the TRXL data even without any consideration for the interdependence. Regarding hydrogen bonding, to check if the classical MD simulations with the OPLS-AA force field, a six-site all-atom (AA) model, can account for hydrogen bonding, we used the MD snapshots to calculate the theoretical static scattering curve of I_3^- in methanol. The calculated curve agrees well with the experimental scattering curve (Supplementary Fig. 15). The six-site AA model is known to reproduce the results from the classical three-site models with UA (united atom model) where identical atoms are treated altogether. The differences between the six-site and the three-site models regarding the intermolecular potential or the molecular structural parameters are negligible compared to those between various other models proposed for methanol. In addition, if we distribute the charge of the methyl group ($-CH_3$) in the three-site model by the C:H ratio of 4:7 in the six-site model, it was reported that the dipole moment of methanol increases by only ~10%, which is still small compared to the electronegativity between C/H and I[19-21]. Thus, the conclusion regarding the charge distribution is unlikely to be affected by the choice of the force field. Nevertheless, one cannot rule out the possibility that the exact global minimum may change if different MD simulations to obtain the cage term coupled with the charge distribution are used instead of the current classical MD simulations with the OPLS-AA force field. The exploration in this regard is beyond the scope of the current study, but the approach outlined in this work should provide a road for such efforts.

Supplementary Note 2: Simulations to quantify the sensitivities of isotropic and anisotropic signals on the shorter-bond and longer-bond dissociations

To ensure that the shorter-bond dissociation and longer-bond dissociation can be distinguished by the TRXL data and to quantify the sensitivities of ΔS_{iso} and ΔS_{aniso} on the two dissociation models, we conducted simulations. For this simulation, we considered that the dissociation process is completed and the anisotropic orientational distributions of I_2^- and the depleted population of I_3^-. The direction of the transition dipole in I_3^- becomes the direction of bond dissociation, and a linearly polarized laser pulse preferentially excites molecules with the transition dipoles oriented along the laser polarization direction with a cosine-square distribution. More specifically, the excited molecules are oriented with a distribution of $\cos^2\phi$, where ϕ is the angle between the laser polarization and the transition dipole. For the simulation, we considered that I_B-I_C of I_3^- is aligned to the laser polarization for the case of the shorter I-I bond dissociation, whereas I_A-I_B is aligned for the case of the longer-bond dissociation. Noticeably, to calculate the ΔS_{aniso} more accurately, we took the anisotropic cage terms of I_3^- and I_2^- into account for the calculation. In the simulations, the structural information of both I_3^- and I_2^- was set at those obtained from structural analysis at 100 ps (R_{AB} = 3.09 Å, R_{BC} = 2.96 Å, θ = 152°, and R(I_2^-) = 3.25 Å). Details on calculating difference scattering curves of isotropic and anisotropic components are described in references[1,2]. Results are shown in Supplementary Fig. 9. Because ΔS_{iso} calculated by the Debye equation is independent of the orientation of the molecules, both models show identical isotropic difference scattering curves (Supplementary Fig. 9a). In contrast, the transition dipole moment of the molecule which is aligned to the polarization axis of the pump laser should be considered to calculate the ΔS_{aniso}. The one-photon excitation process induces the cosine square distribution along the laser polarization axis of the target molecules, and the direction of dipole moment determines the angular distribution of the excited and

unexcited molecules. Thus, we calculated the ΔS_{aniso} assuming that R_{BC} and R_{AB} are parallel to the transition dipole moment of I_3^- for the shorter-bond dissociation model and the longer-bond dissociation model, respectively. Calculated ΔS_{aniso} of each model is presented in Supplementary Fig. 9b. For calculating the anisotropic difference curves, anisotropy coefficients of I_3^- and I_2^- were set to be unity. The simulation results show that the ΔS_{aniso} curves from two dissociation models show a noticeable difference in several q regions, around 3, 4, and 6 Å^{-1}. It should be noted that the structural information used for each dissociation model is the same for both models. Thus, we can conclude that the molecular alignment during the photoexcitation process, especially using the linearly polarized pump laser, can be resolved using the anisotropic signal of solution scattering signal.

Supplementary Table

Table S1. Structural parameters of I_3^- and I_2^- optimized from the structural fitting analysis.

(i) The final optimized structure, which is asymmetric and bent and self-consistent with the cage term, (I_A, I_B, I_C) = (-0.9 e, 0.0 e, -0.1 e)

wR		0.0681	
wR_{free}		0.0683	
Intermediate	Parameter	Fit value	Error
I_3^-	R_{AB} (Å)	3.09	0.01
	R_{BC} (Å)	2.96	0.01
	I-I-I angle (°)	152.0	0.4
I_2^-	$R(I_2^-)$ (Å)	3.25	0.01

(ii) Optimized structure using the initial guess of a symmetric linear structure used to calculate the cage term, (I_A, I_B, I_C) = (-0.5 e, 0.0 e, -0.5 e)

wR		0.0804	
wR_{free}		0.0831	
Intermediate	Parameter	Fit value	Error
I_3^-	R_{AB} (Å)	3.14	0.01
	R_{BC} (Å)	2.92	0.01
	I-I-I angle (°)	151.2	0.3
I_2^-	$R(I_2^-)$ (Å)	3.28	0.01

(iii) Optimized structure, which is bent and asymmetric and self-consistent with the cage term, (I_A, I_B, I_C) = (0.0 e, -1.0 e, 0.0 e)

wR		0.1228	
wR_{free}		0.1356	
Intermediate	Parameter	Fit value	Error
I_3^-	R_{AB} (Å)	3.14	0.01
	R_{BC} (Å)	2.97	0.01
	I-I-I angle (°)	148.5	0.3
I_2^-	$R(I_2^-)$ (Å)	3.31	0.01

Supplementary Figures

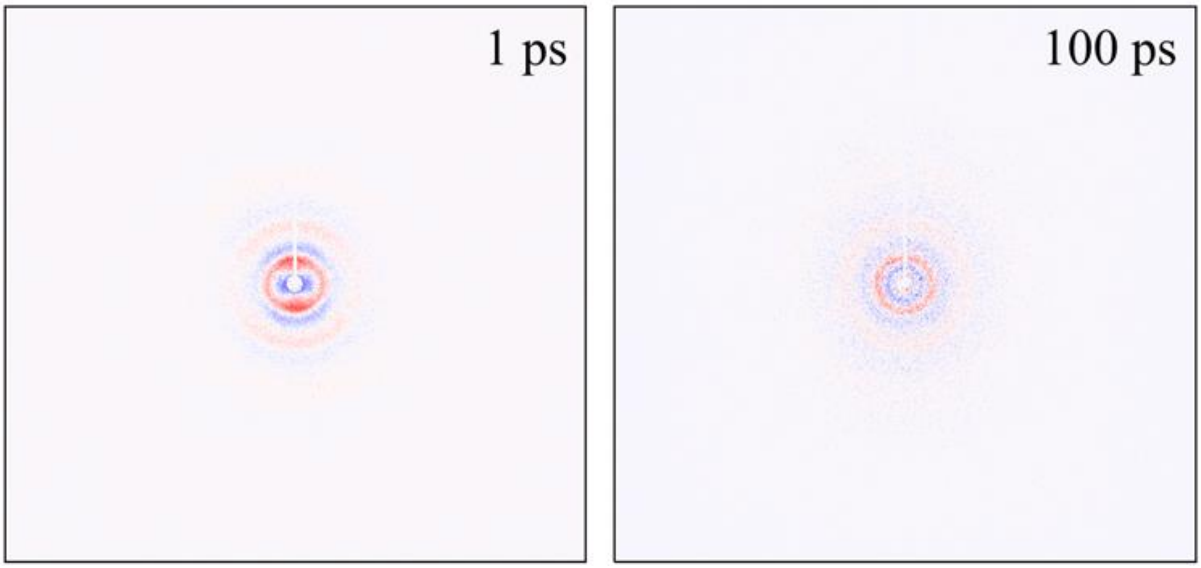

Supplementary Fig. 1. Raw 2D difference x-ray scattering images at two representative time delays. The experimental 2D difference scattering images at 1 ps (left) and 100 ps (right) are shown for comparison. The former and latter are anisotropic and isotropic, respectively.

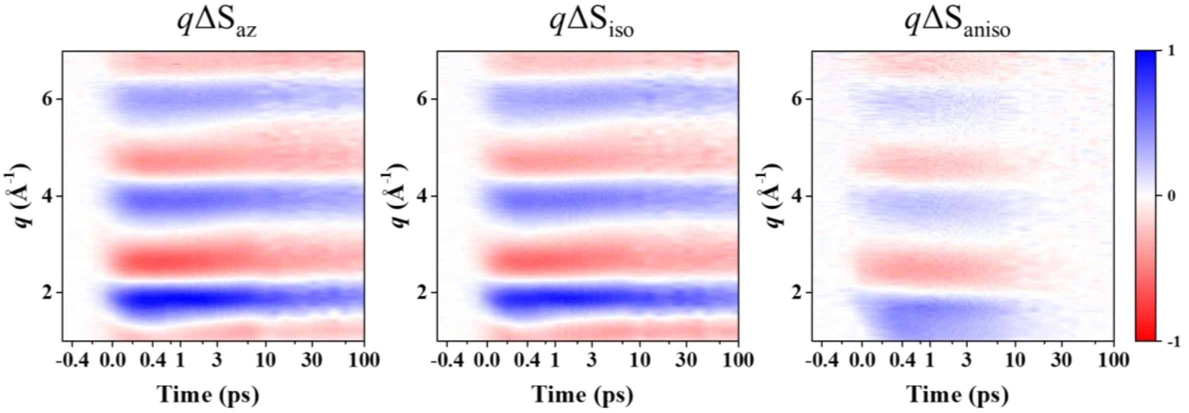

Supplementary Fig. 2. Experimental difference scattering curves. The $q\Delta S_{az}$, $q\Delta S_{iso}$, and $q\Delta S_{aniso}$ curves from fs-TRXL data on I_3^- in methanol are shown. All plots share a color scale representing the amplitude of the signal in absolute electronic units per solvent molecule.

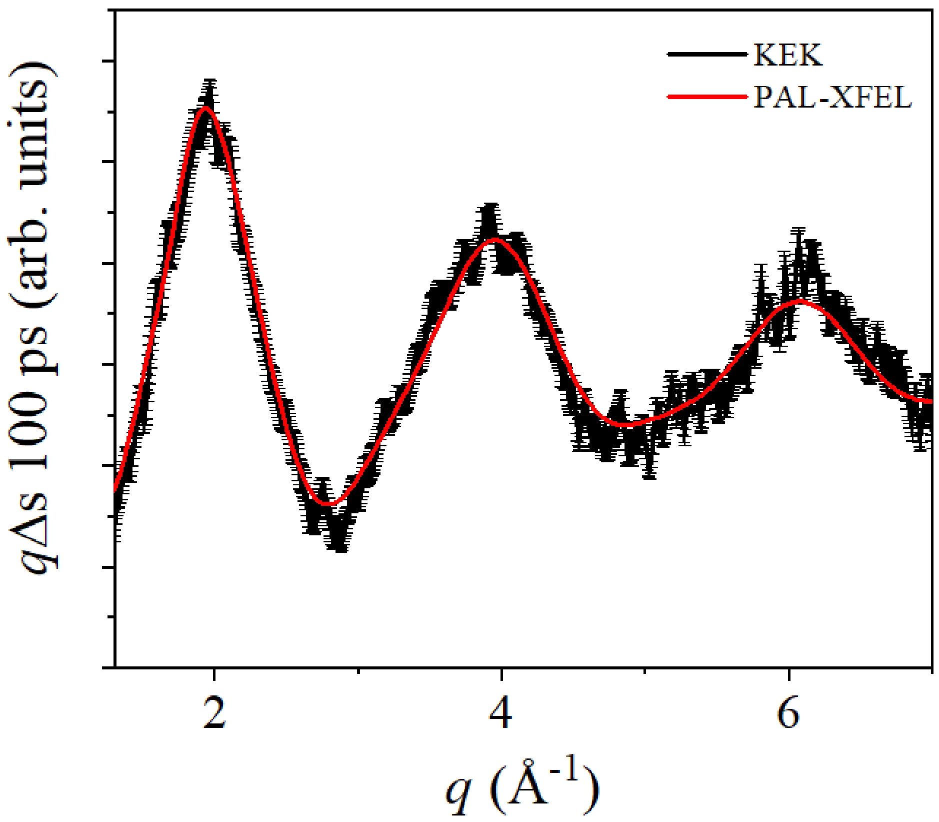

Supplementary Fig. 3. Comparison of the data of this work with that of previous work. The experimental 100 ps data measured at KEK from the previous study (black) with the corresponding standard error as error bars[4] and the 100 ps data of this study measured at PAL-XFEL (red) are compared. For comparison, a polychromatic correction was applied to the PAL-XFEL data because the data at KEK used a polychromatic x-ray beam with a broad bandwidth.

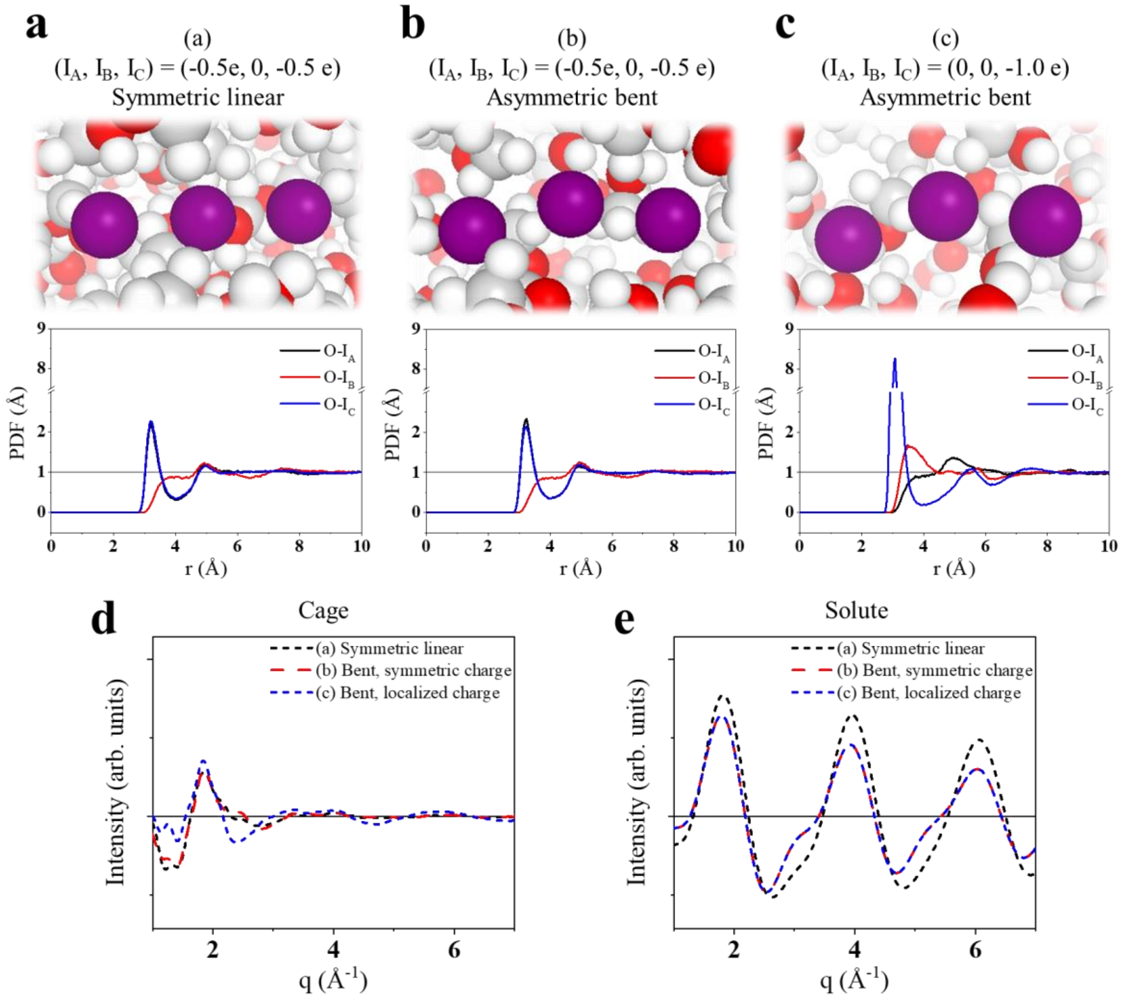

Supplementary Fig. 4. The effect of charge distribution on the cage term and the results of the grid searching with a fixed cage structure. (**a-c**) MD simulations of various conformations and charge distributions were conducted, and the resulting PDFs of the oxygen of methanol and each I atom of I_3^- are shown for three cases: (a) the structure and charge distribution of I_3^- calculated by DFT calculation was used, (b) the initial structure from the structural fitting process with a symmetric charge distribution where the charge is shared by two terminal iodine atoms was used, and (c) the initial structure from the structural fitting process with the localized negative charge on the terminal I atom (I_C) was used. (**d-e**) The corresponding (d) cage terms and (e) solute terms are displayed. The PDFs in (a) – (c) and the cage terms in (d) show that the change in charge distribution induces a dramatic change in the cage structure.

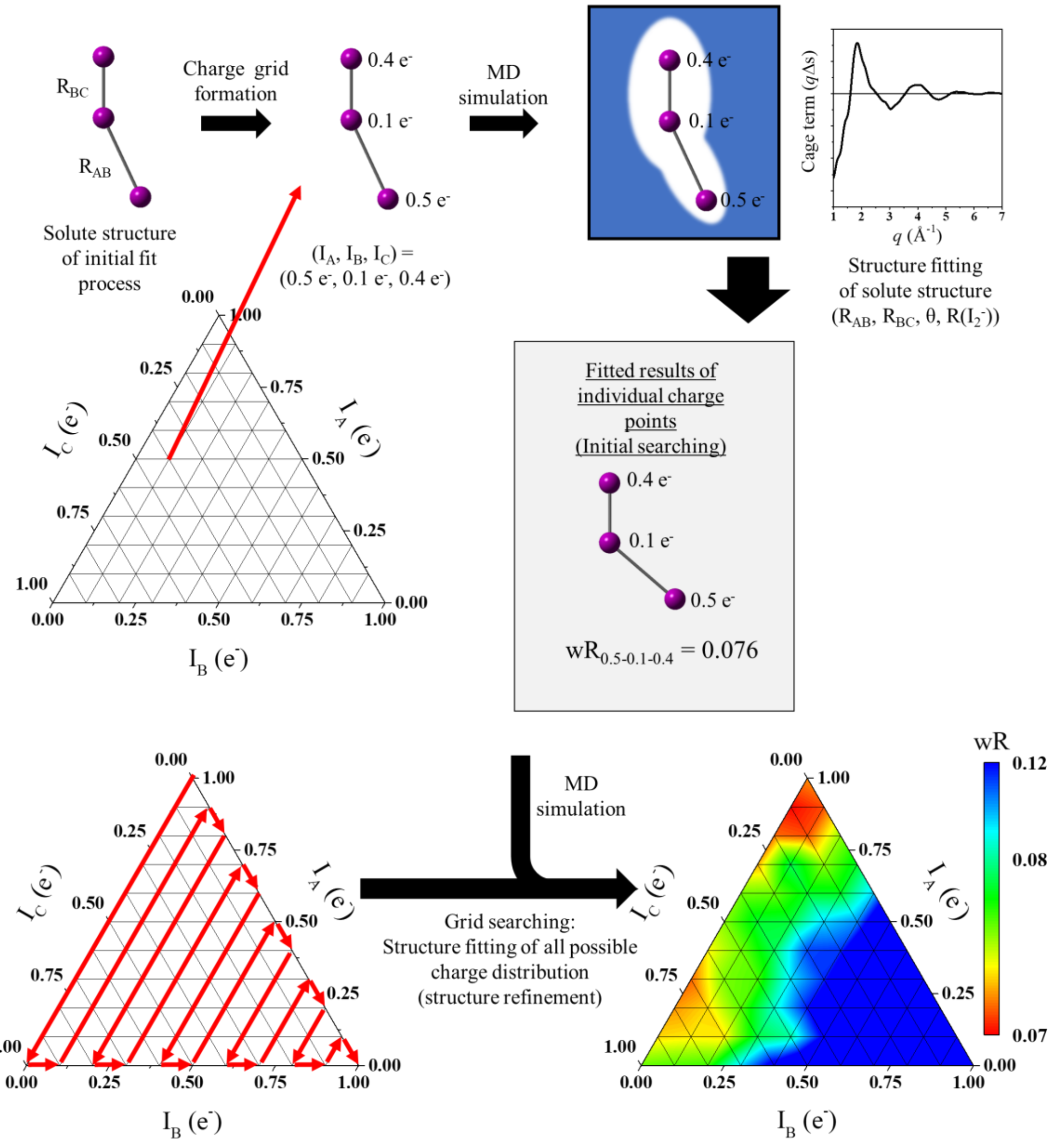

Supplementary Fig. 5. The procedure for grid search analysis to verify the charge distribution. For the grid search analysis, two search processes, the initial grid searching and the structure refinement at each grid point, were conducted. For the first step (initial grid searching), initial charge grid points and the corresponding cage term at each grid point were prepared using the structural information determined from the initial fitting process (R_{AB} = 3.14 Å, R_{BC} = 2.92 Å, θ = 151.2°). From the results, we can get the fitted results of I_3^- and I_2^- which were used to calculate new cage terms for structure refinement. For the second step (structure refinement), the cage term at each grid point was calculated using the structural

information extracted from the previous step. From this two-step grid searching process, we obtained the refined grid searching results.

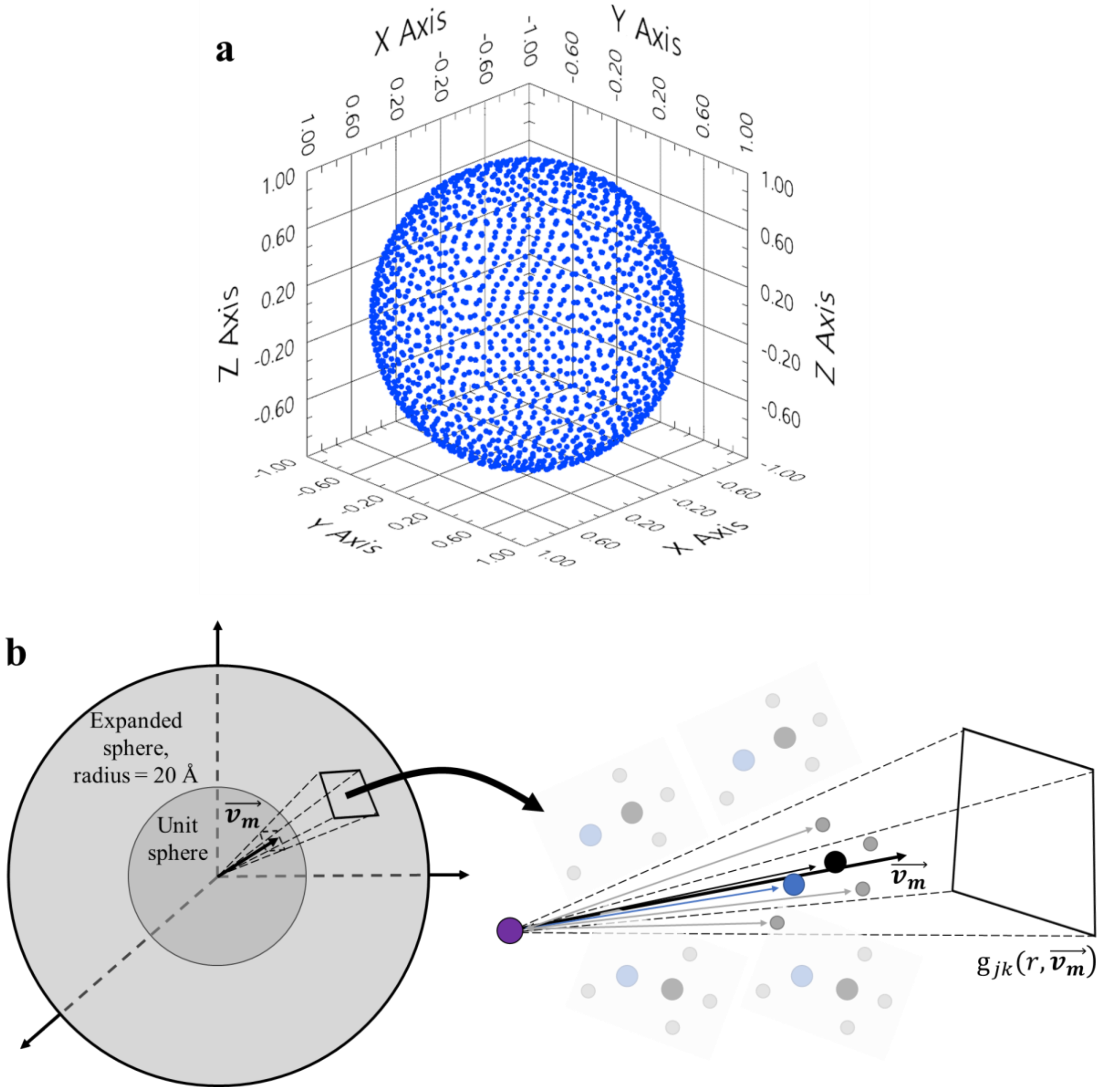

Supplementary Fig. 6. Methodology to construct the orientation-dependent PDF. (**a**) The evenly-spaced unit angular vectors devised to describe the anisotropic distribution of solvent used for calculating anisotropic cage term are indicated as blue dots. The unit angular vectors form a unit sphere spanning the space around the solute atom and are used to calculate orientation-dependent PDFs. (**b**) With the evenly-spaced unit angular vectors, we first generated a new sphere by expanding the unit sphere so that the MD box can be included inside the expanded sphere. The expanded sphere with a radius of 20 Å was enough to cover the MD box. This expanded sphere around a solute molecule is evenly divided into polyhedral cones, each of which has an equal solid angle and has a direction specified by a

unit angular vector. To calculate the orientation-dependent PDF for an MD snapshot (for example, $g_{jk}^{(i)}(r,\overrightarrow{\boldsymbol{v_m}})$ for i-th MD snapshot), we identified the desired atoms (for example, k) of solvent molecules within the polyhedral cone of the expanded sphere and calculated the PDF for the cross pair of an atom of a solute molecule (for example, j) and the selected solvent atoms within each polyhedral cone specified by $\overrightarrow{\boldsymbol{v_m}}$.

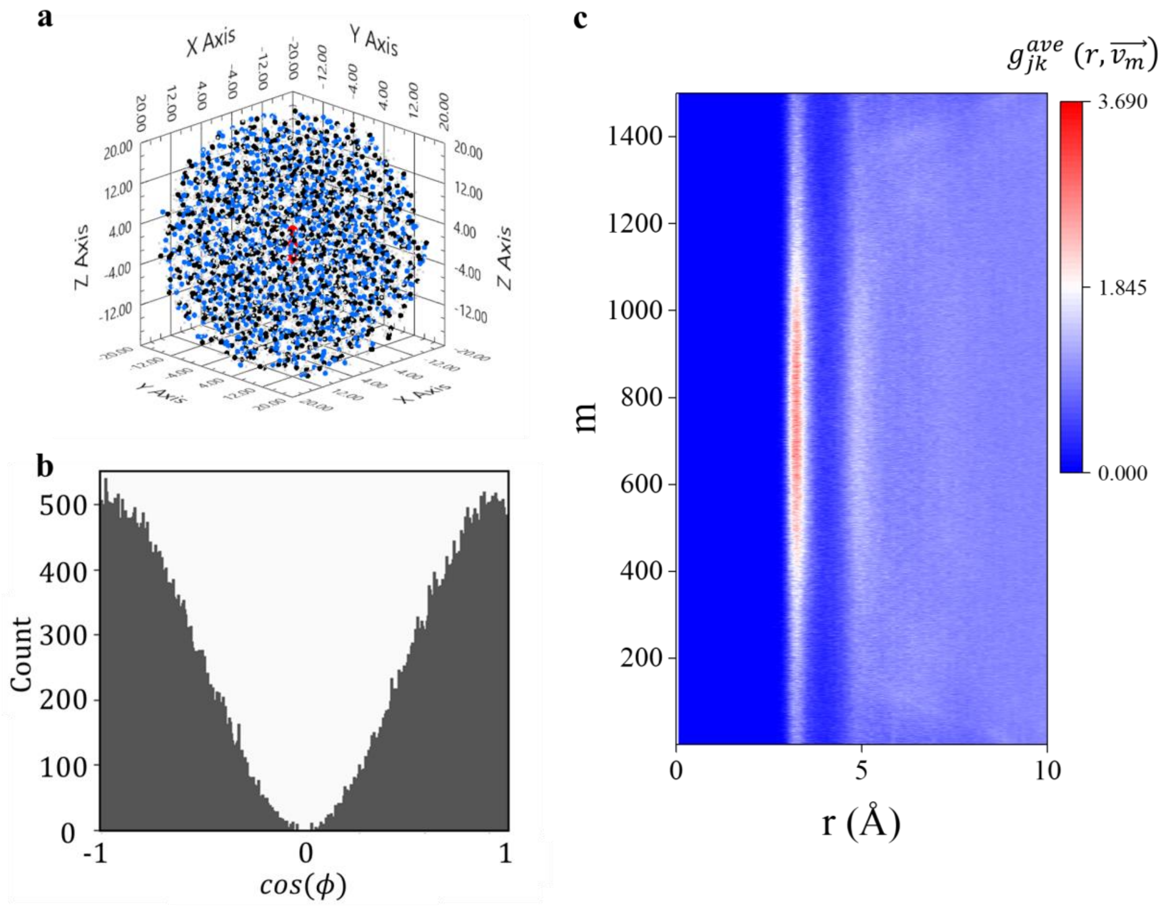

Supplementary Fig. 7. Generating $\cos^2\phi$ distribution to calculate the anisotropic cage terms. (**a**) One of MD snapshots used for calculating anisotropic cage terms is shown (red: iodine, blue: oxygen, black: carbon, light gray: hydrogen). As a first step for calculating an anisotropic cage term, 200,000 MD snapshots were rotated so that the rotated solute molecules have the distribution of $P(\cos\phi) \propto \cos^2\phi$. (**b**) The population of orientations of the rotated MD snapshots as a function of $\cos\phi$ is shown. This population can be approximated as the $\cos^2\phi$ distribution, confirming that the rotation targeting the desired $\cos^2\phi$ distribution worked well. (**c**) For each rotated MD snapshot, the corresponding orientation-dependent PDFs, $g_{jk}^{(i)}(r, \overrightarrow{\boldsymbol{v_m}})$, was calculated, and the resulting 200,000 orientation-dependent PDFs were averaged to yield the averaged orientation-dependent PDFs, $g_{jk}^{ave}(r, \overrightarrow{\boldsymbol{v_m}})$. The averaged orientation-dependent PDFs of the I_A-O pair obtained from MD

snapshots for linear symmetric I_3^- are shown as an example.

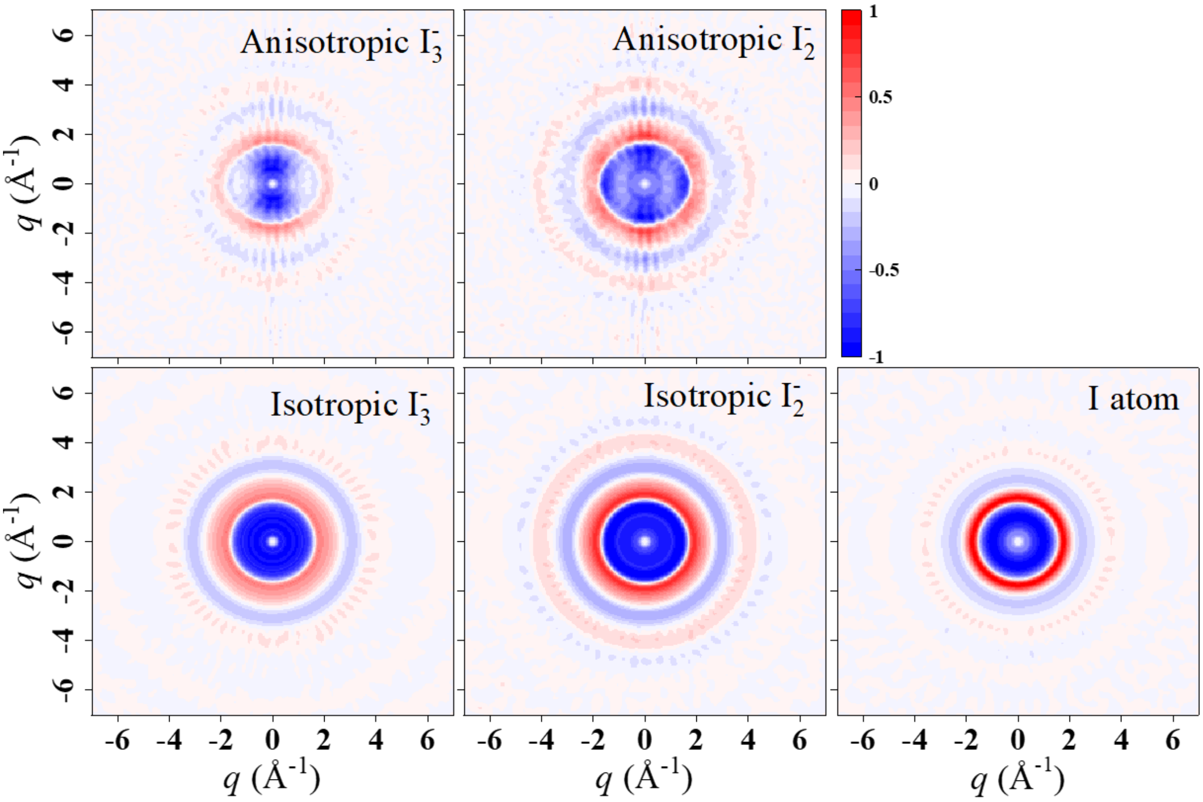

Supplementary Fig. 8 Calculated cage scattering patterns using Eq. (S22). All plots share a color scale representing the amplitude of the signal in absolute electronic units per solvent molecule.

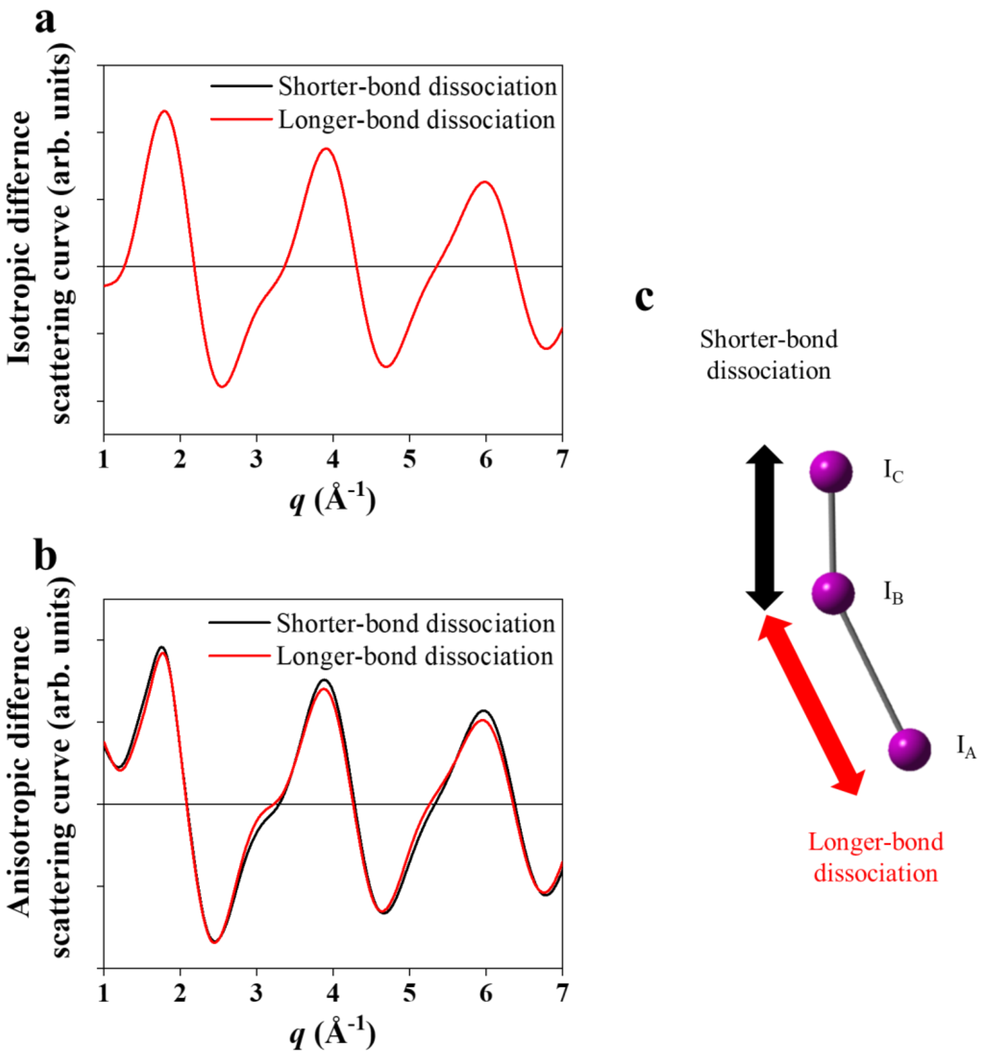

Supplementary Fig. 9. Effect of photoselective alignment and the direction of bond cleavage on the isotropic and anisotropic scattering signals. (**a**) The theoretical $q\Delta S_{iso}$ for two dissociation models are compared. Since there is no difference between shorter-bond dissociation (I_B-I_C aligned) and longer-bond dissociation (I_A-I_B aligned) models in calculating isotropic signal, the calculated results show two identical $q\Delta S_{iso}$. (**b**) The theoretical $q\Delta S_{aniso}$ for two dissociation models are compared. Two different $q\Delta S_{aniso}$ were calculated using identical molecules with different alignment along the laser polarization axis. (**c**) The alignment directions for two dissociation models are indicated with the black (shorter-bond dissociation) and the red (longer-bond dissociation) arrows.

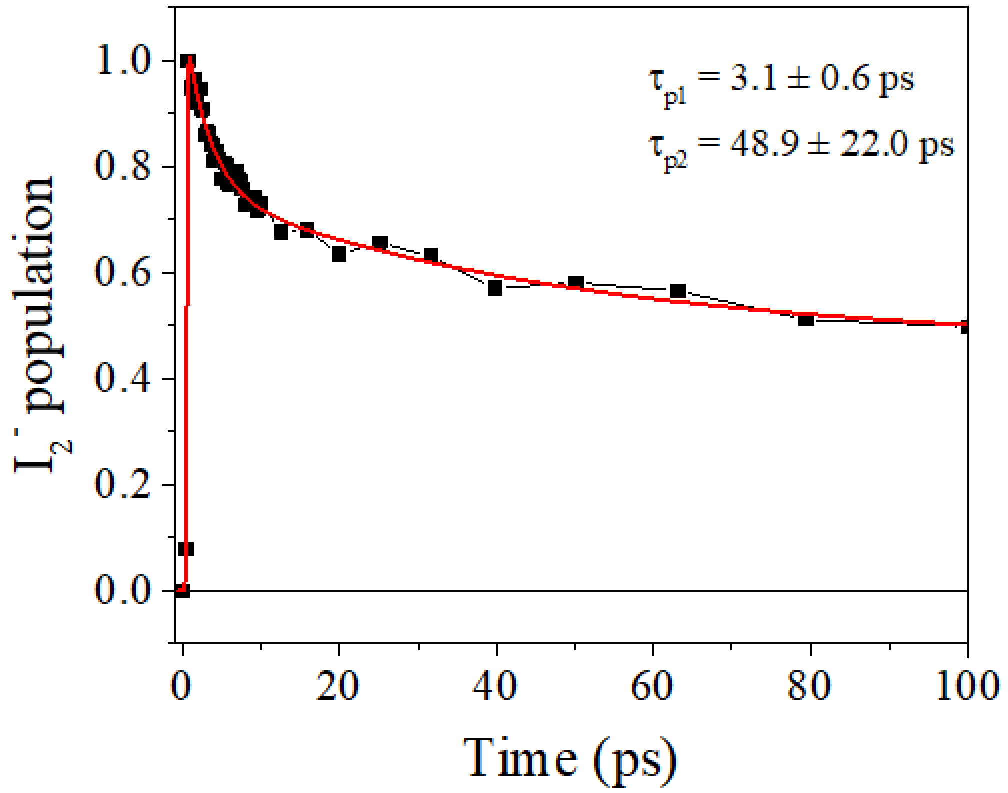

Supplementary Fig. 10. The population change of I_2^-. The population change of the I_2^- determined from the fitting of the dataset, which spans -2 ps to 100 ps (black dot). The time-dependent population change of I_2^- was fitted with a double-exponential function convoluted with the IRF (instrument response function) of a Gaussian function. The width of IRF was determined from the rSV fitting results (0.27 ps, FWHM).

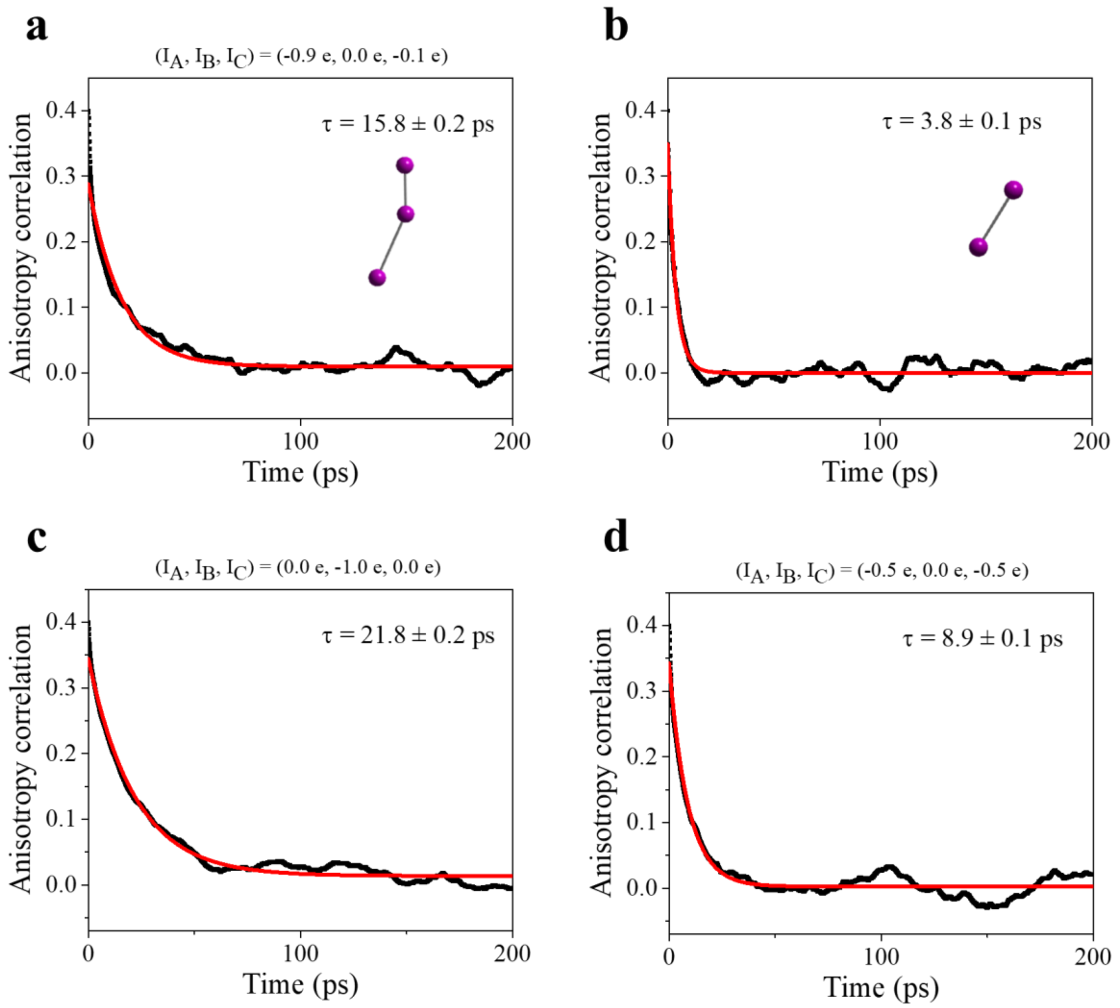

Supplementary Fig. 11. The calculated rotational correlation function of I_3^- and I_2^- by the MD simulation. Rotational correlation functions (RCF) of solute molecules are calculated by the MD simulation. RCF was calculated at the equilibrium-state molecules by averaging the RCF of each MD snapshot. For each case, the RCF from simulation (black) and the exponential fit (red) are presented. (**a**) The RCF of I_3^- that has the optimized structure and charge distribution is shown. The fitted curve gives a rotational diffusion time constant of 15.8 ± 0.2 ps. (**b**) The RCF of I_2^- is shown. The fitted curve gives a rotational diffusion time constant of 3.8 ± 0.1 ps. (**c**) The RCF of I_3^- which has the localized charge on the central iodine atom is shown. The fitted curve gives a time constant of 21.8 ± 0.2 ps. (**d**) The RCF of I_3^- where the charge is equally distributed on both terminal iodine atoms is shown. The fitted

curve gives a time constant of 8.9 ± 0.1 ps time constant.

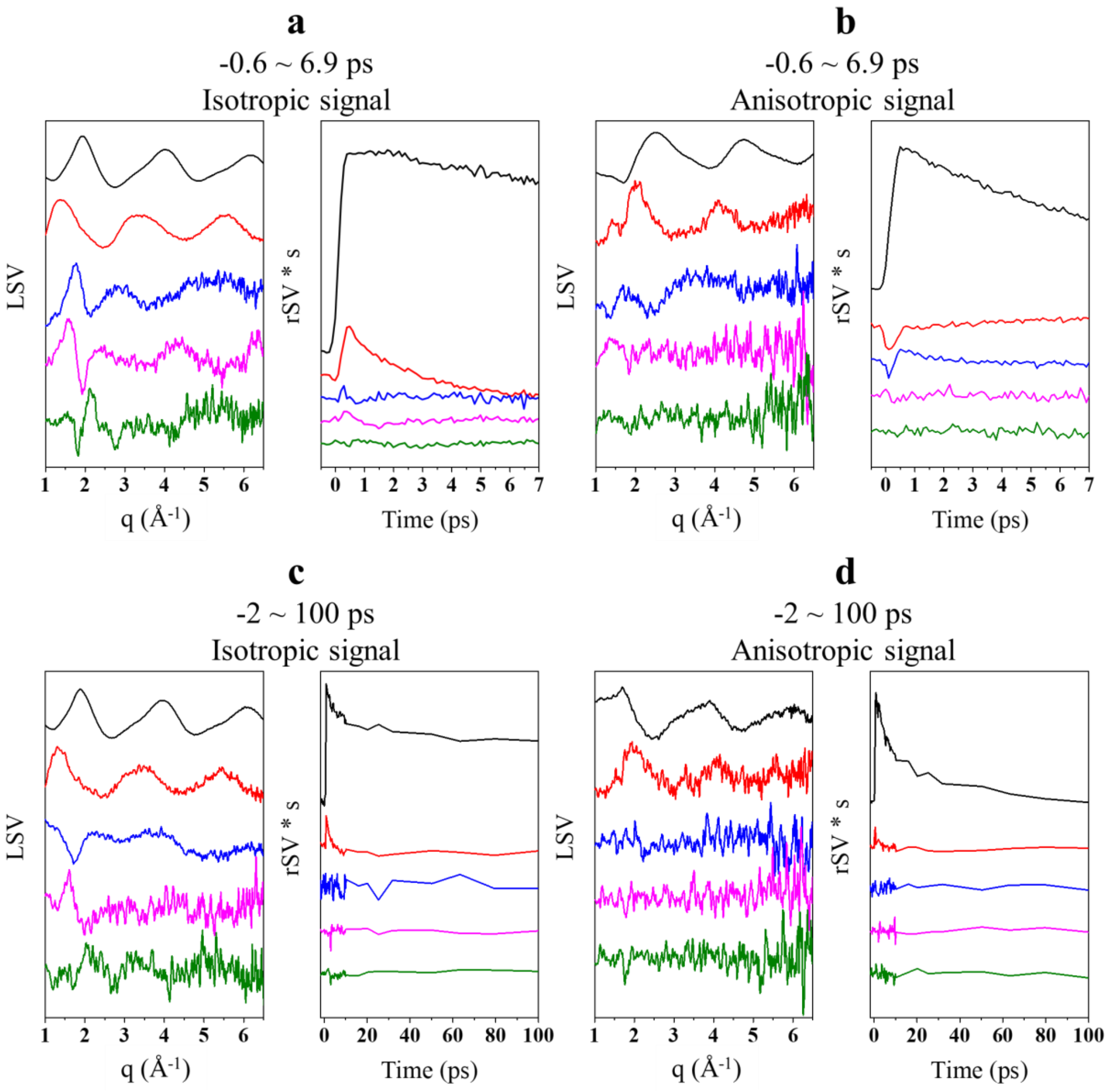

Supplementary Fig. 12. SVD results. The SVD results of the (**a**) isotropic and (**b**) anisotropic $q\Delta S(q,t)$ at the t = -0.6 ~ 6.9 ps region show the early time dynamics. The SVD results of the (**c**) isotropic and (**d**) anisotropic $q\Delta S(q,t)$ data at the t = -2 ~ 100 ps region show late time delay dynamics. In each figure, left singular vectors and right singular vectors multiplied by the corresponding singular values are shown in the left and right panels, respectively.

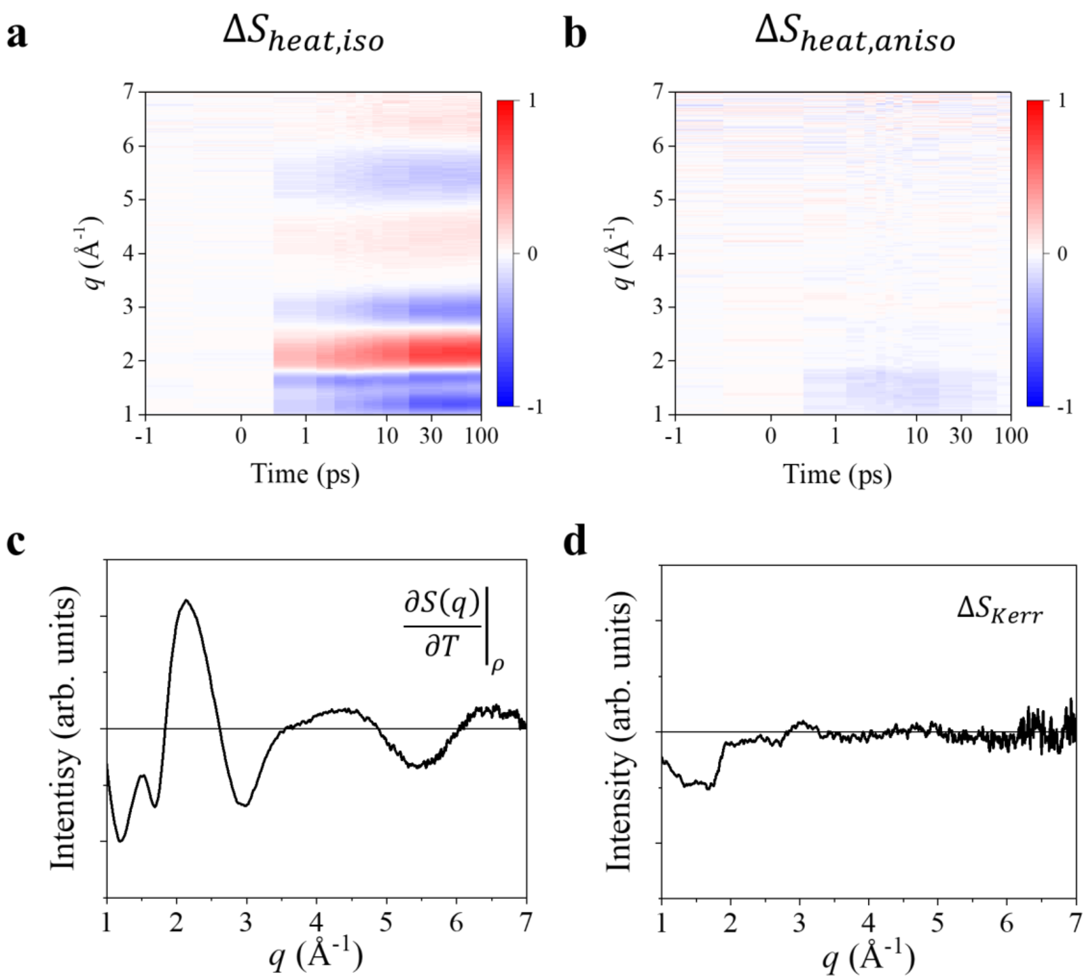

Supplementary Fig. 13. Experimentally measured isotropic and anisotropic heating signals. For the fitting process, we experimentally measured the heating signal using a heating dye. (**a**) The obtained isotropic heating signal, $\Delta S_{heat,iso}$, and (**b**) the anisotropic heating signal, $\Delta S_{heat,aniso}$, are presented. (**c**) Because the analyzed time delays in our study are within 100 ps where the thermal expansion does not occur yet, the main contribution of the solvent heating process is the temperature solvent differential, $\left.\frac{\partial S(q)}{\partial T}\right|_{\rho}$, which was obtained by taking the signal at 100 ps. (**d**) We extracted ΔS_{Kerr} from the $\Delta S_{heat,aniso}$ by taking the 1st lSV of the SVD result.

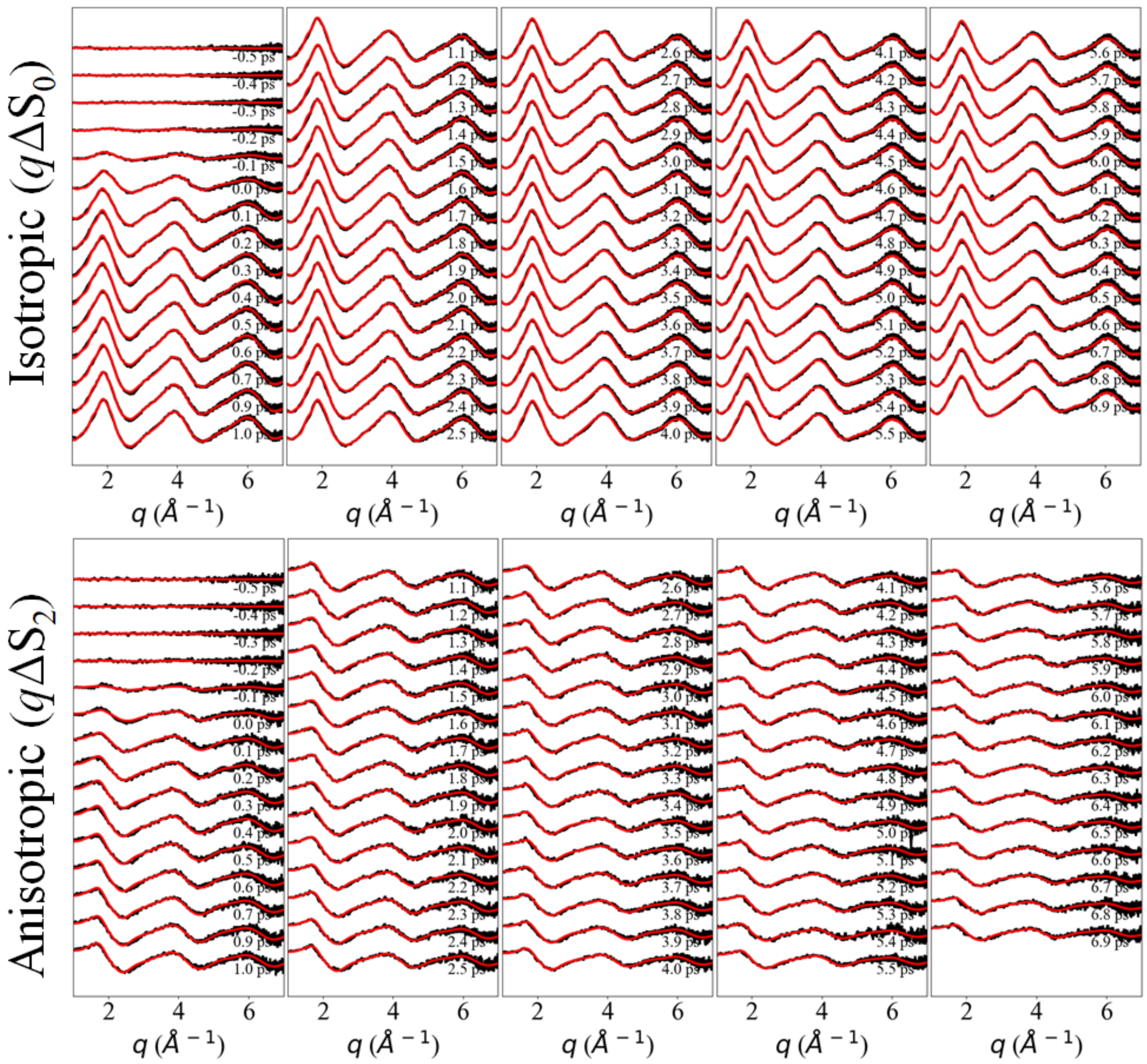

Supplementary Fig. 14. Fit results for the isotropic and anisotropic difference scattering curves of the I_3^- solution. Experimental curves with the corresponding standard error as error bars and the theoretical curves are shown in black and red lines, respectively.

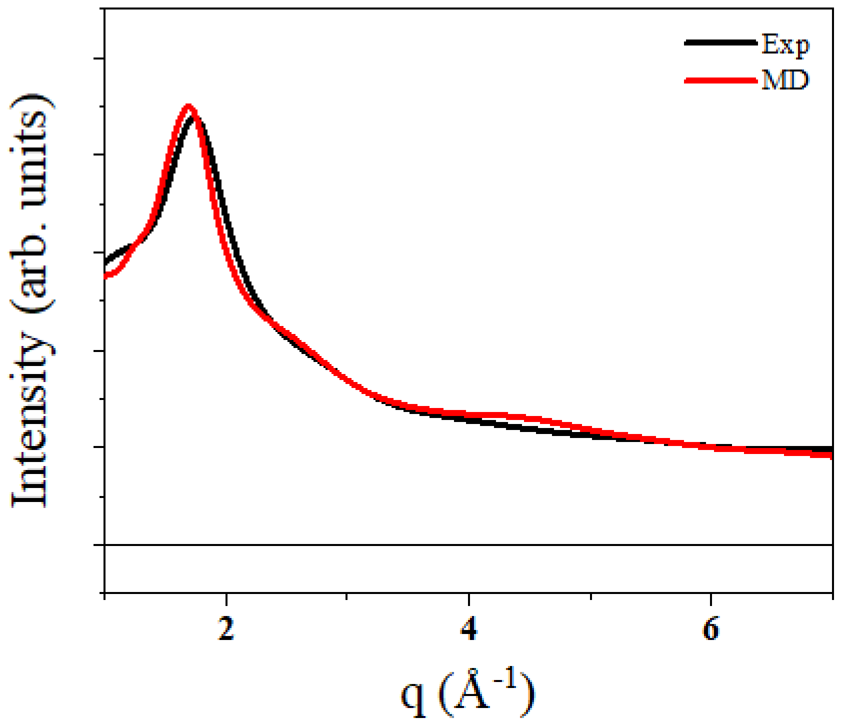

Supplementary Fig. 15. Comparison between the experimental static scattering curve and the calculated static scattering curve from the MD simulation.

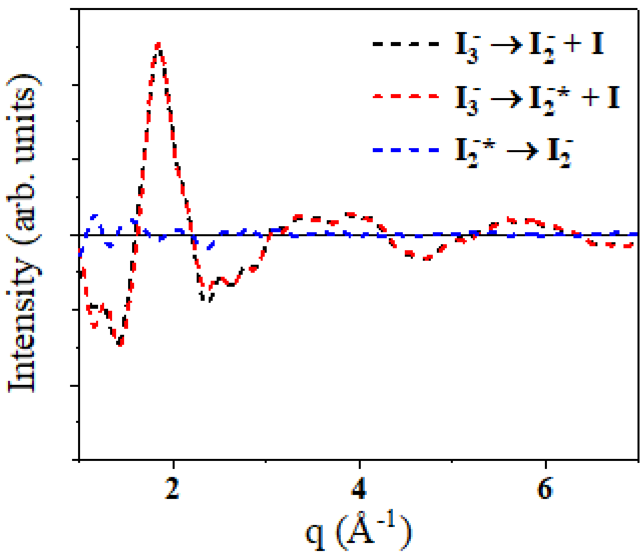

Supplementary Fig. 16 Comparison of the cage terms for I_3^- → I_2^- + I and I_3^- → I_2^{-*} + I. The cage term for I_3^- → I_2^- + I is shown in black and that for I_3^- → I_2^{-*} + I is shown in red. Their difference, which corresponds to the cage for I_2^{-*} → I_2^-, is shown in blue. I_2^{-*} and I_2^- indicate the vibrationally excited diiodide (R(I_2^-) = 3.34 Å) and vibrationally relaxed diiodide (R(I_2^-) = 3.25 Å), respectively.

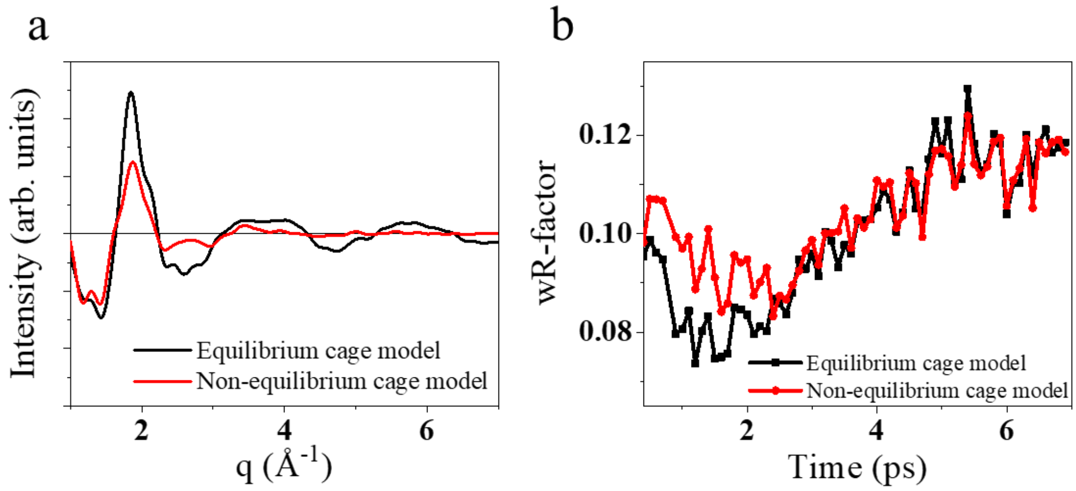

Supplementary Fig. 17 Comparison of the fitting results using two different cage models. (a) The cage terms of the equilibrium cage model and the non-equilibrium cage model are presented. (b) The wR-factors of two cage models are shown. The equilibrium cage model shows smaller wR-factors at early time delays.

Supplementary References

1 Biasin, E. *et al.* Anisotropy enhanced X-ray scattering from solvated transition metal complexes. *J. Synchrotron Radiat.* **25**, 306-315 (2018).

2 Baskin, J. S. & Zewail, A. H. Oriented ensembles in ultrafast electron diffraction. *Chemphyschem* **7**, 1562-1574 (2006).

3 Kim, K. H. *et al.* Global reaction pathways in the photodissociation of I3 (-) ions in solution at 267 and 400 nm studied by picosecond X-ray liquidography. *Chemphyschem* **14**, 3687-3697 (2013).

4 Kim, K. H. *et al.* Solvent-dependent molecular structure of ionic species directly measured by ultrafast x-ray solution scattering. *Phys. Rev. Lett.* **110**, 165505 (2013).

5 Josefsson, I. *et al.* Collective hydrogen-bond dynamics dictates the electronic structure of aqueous I3(-). *Phys. Chem. Chem. Phys.* **15**, 20189-20196 (2013).

6 Jena, N. K. *et al.* Solvent-dependent structure of the I3(-) ion derived from photoelectron spectroscopy and ab initio molecular dynamics simulations. *Chemistry* **21**, 4049-4055 (2015).

7 Berntsson, O. *et al.* Time-Resolved X-Ray Solution Scattering Reveals the Structural Photoactivation of a Light-Oxygen-Voltage Photoreceptor. *Structure* **25**, 933-938.e933 (2017).

8 Ravishankar, H. *et al.* Tracking Ca(2+) ATPase intermediates in real time by x-ray solution scattering. *Sci. Adv.* **6**, eaaz0981 (2020).

9 Kim, T. K., Lee, J. H., Wulff, M., Kong, Q. & Ihee, H. Spatiotemporal kinetics in solution studied by time-resolved X-ray liquidography (solution scattering). *Chemphyschem* **10**, 1958-1980 (2009).

10 James, F. & Roos, M. Minuit - a system for function minimization and analysis of the parameter errors and correlations. *Comput. Phys. Commun.* **10**, 343-367 (1975).

11 Kjær, K. S. *et al.* Introducing a standard method for experimental determination of the solvent response in laser pump, X-ray probe time-resolved wide-angle X-ray scattering experiments on systems in solution. *Phys. Chem. Chem. Phys.* **15**, 15003-15016 (2013).

12 Ihee, H. *et al.* Ultrafast x-ray diffraction of transient molecular structures in solution. *Science* **309**, 1223-1227 (2005).

13 Cammarata, M. *et al.* Impulsive solvent heating probed by picosecond x-ray diffraction. *J. Chem. Phys.* **124**, 124504 (2006).

14 Zhang, X.-X., Breffke, J., Ernsting, N. P. & Maroncelli, M. Observations of probe dependence of the solvation dynamics in ionic liquids. *Phys. Chem. Chem. Phys.* **17**, 12949-12956 (2015).

15 Kim, J. G. *et al.* Mapping the emergence of molecular vibrations mediating bond formation. *Nature* **582**, 520-524 (2020).

16 Ki, H., Lee, Y., Choi, E. H., Lee, S. & Ihee, H. SVD-aided non-orthogonal decomposition (SANOD) method to exploit prior knowledge of spectral components in the analysis of time-resolved data. *Struct. Dyn.* **6**, 024303 (2019).

17 Guinier, A. *X-ray diffraction in crystals, imperfect crystals, and amorphous bodies*. (W.H. Freeman, 1963).

18 Koay, C. G. Analytically exact spiral scheme for generating uniformly distributed points on the unit sphere. *J. Comput. Sci.* **2**, 88-91 (2011).

19 Haughney, M., Ferrario, M. & McDonald, I. R. Molecular-dynamics simulation of liquid methanol. *J. Phys. Chem.* **91**, 4934-4940 (1987).

20 Hawlicka, E., Palinkas, G. & Heinzinger, K. A molecular dynamics study of liquid methanol with a flexible six-site model. *Chem. Phys. Lett.* **154**, 255-259 (1989).
21 Shilov, I. Y., Rode, B. M. & Durov, V. A. Long range order and hydrogen bonding in liquid methanol: A Monte Carlo simulation. *Chem. Phys.* **241**, 75-82 (1999).
